# Functional variants of CFAP410 affect the DNA damage response leading to motor neuron degeneration – Implications for ALS

**DOI:** 10.1016/j.isci.2025.113338

**Published:** 2025-08-09

**Authors:** Ross Ferguson, Vasanta Subramanian

**Affiliations:** 1 Department of Biology & Biochemistry, University of Bath, Claverton Down, BA2 7AY Bath, UK

**Keywords:** Molecular biology, Neuroscience, Cell biology

## Abstract

Mutations in CFAP410, a basal body protein known to be required for the formation of primary cilia, have been identified as risk modifiers in amyotrophic lateral sclerosis (ALS), a devastating late onset neurodegenerative disorder with poor prognosis. CFAP410 is also implicated in the DNA damage response and interacts with Nek1, which has been shown to be mutated in ALS. Herein, we investigated the effect of knocking in an HA epitope tag and functional mutations into the endogenous Cfap410 gene by gene editing in mouse embryonic stem cells (mESCs). We show that primary cilia in these edited mESCs, as well as in the neural progenitors and neurons differentiated from them do not exhibit any significant difference in frequency. However, ESCs, neural progenitors, and neurons with knock-in Cfap410 variants are more susceptible to DNA damage and exhibit impaired interaction with Nek1. Our findings point to DNA damage as a convergent pathway leading to ALS.

## Highlights

• Endogenously epitope tagged Cfap410 variants in mouse embryonic stem cells were created
• Tagged V20M and R73P variants identified in human populations localize to primary cilia
• Show reduced interactions with the DNA damage response protein in motor neurons
• Impaired DNA damage response in ESC, neural progenitors, and motor neurons

## Graphical abstract

**Figure:**
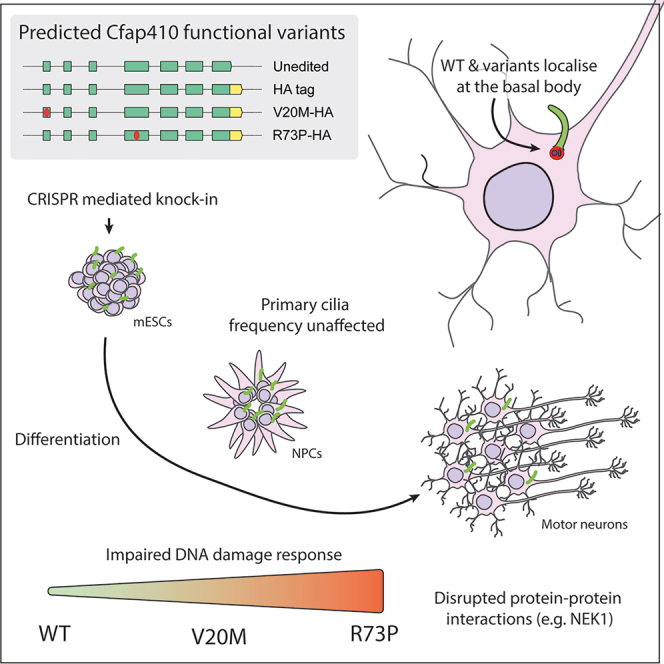

## Introduction

Amyotrophic lateral sclerosis (ALS) is a fatal neurodegenerative disorder affecting upper and lower motor neurons with a prevalence of between 3 and 5 per 100 000 persons worldwide, and this incidence is on the increase.^1^^,^^2^^,^^3^ Several new causative/risk genes have been/are being identified for ALS,^4^^,^^5^ and one of these is the Cilia and Flagella associated protein 410 (*CFAP410*- alias *C21orf2)*.^6^ Besides ALS, mutations in *CFAP410* have also been reported in ciliopathies such as Axial spondylometaphyseal dysplasia, Jeune syndrome and retinal dystrophy.^7^^,^^8^^,^^9^

The biological function of CFAP410 is not particularly well understood. It appears to be essential for primary cilia formation^7^^,^^8^^,^^10^ and has also been shown to play a role in the organization of the actin cytoskeleton.^11^ Immuno-histochemical studies in human, pig, and mouse retinas localized the CFAP410 protein to the ciliary structures of the photoreceptor cells.^12^ The expression of *CFAP410* is also seen in the brain and spinal cord (FANTOM5 and MGI Gene Expression Database) and shown to localize to mitochondria in immune cells. The expression of *CFAP410* in the brain and spinal cord and the occurrence of variants seen in ALS suggest that *CFAP410* has an important role to play in the nervous system. CFAP410 is also likely to be a part of the ciliary structures of cells in the brain.

Primary cilia play a key role in transducing signals, particularly of the sonic hedgehog signaling pathway. This pathway plays a key role in the normal development of the central nervous system, particularly the hippocampus, cerebellum, and spinal cord.^13^ Loss of *CFAP410* in siRNA-treated IMCD3 cells led to either complete loss of cilia and/or a few cilia reduced to stumps (≤1.5 μm)^10^ while complete knock-out reduced primary cilia frequency by 60% in ARPE19 cells.^14^ Thus, it appears that CFAP410 is required for the formation and elongation of primary cilia as well as for Shh signaling.

CFAP410 interacts and forms a functional complex with never in mitosis gene A related kinase 1 (NEK1).^7^ NEK1 ALS risk variants have been reported in both familial and sporadic cases of ALS.^15^^,^^16^^,^^17^ NEK1 possesses both serine threonine and tyrosine kinase activities and plays an important role in the regulation of the cell cycle and DNA damage repair.^18^^,^^19^^,^^20^ An ALS variant in this gene leads to increased DNA damage in patient derived motor neurons.^21^ Like NEK1, CFAP410 has also been implicated in DNA repair mechanisms^22^ and more recently the V58L ALS variant of CFAP410 has been shown to be stabilized upon hyperphosphorylation by NEK1^23^ and lead to increased apoptosis and altered DNA damage response in human motor neurons.^24^ Taken together, it is likely that the ALS variants seen in the conserved domains of CFAP410 such as the LRR domains (protein interaction domains) may affect primary cilia and/or the actin cytoskeleton thereby affecting neurons. Furthermore, because of its role in the DNA repair mechanism, CFAP410 ALS variants neurons may exhibit an aberrant DNA damage response.

The variants in CFAP410 associated with ALS occur throughout the protein, and many of these are rare variants.^6^ At present it is unclear which of these are risk variants relevant to the etiology of ALS. A comparison of the amino acid sequence of CFAP410 reveals that there is significant conservation across species. There are two major regions of conservation (1) an N-terminal conserved region (1–142 aa, coded by exons 1–5) where there is a predicted mitochondria localization signal peptide, two tandem leucine-rich repeat 4 (LRR4, residues 40–80) domains followed by a leucine-rich repeat cap (LRR cap, residues 104–122) and (2) a short conserved stretch at the C-terminus (214–256 aa, encoded by exon 7). Neither the C-terminal conserved region nor the variable region exhibits any homology to known domains and proteins.

Using a bioinformatics and structure homology modeling approach we have previously predicted changes in local interactions for the different ALS risk variants of conserved residues of CFAP410.^25^ Here in this report, we have chosen to study the effects of two of these variants predicted to have functional consequences, V20M (dbSNP: rs1014370765) and R73P (dbSNP: rs140451304), on neuroepithelial cells and motor neurons. Modeling suggests the R73P mutation results in loss of interactions with S53 and increased rigidity around the position resulting from the proline. R73P is within the same LRR4 motif as V58L, so could also potentially affect similar protein-protein interactions. The R73P variant has previously been associated with the ciliopathies axial spondylometaphyseal dysplasia and Jeune syndrome.^8^^,^^26^ V20M is a hydrophobic to polar change and predicted to have little change in hydrogen bonding, though it is within the predicted mitochondrial localisation motif. It is found in both case and control populations in Dutch cohorts.^6^ Though the occurrence rate of mutations in CFAP410 suggests it is unlikely to be the primary driver of ALS, we sought to characterize these variants as potential risk alleles based on these predictions, by assaying their effects on motor neurons and further cell types.

In order to do this, we have used a gene editing approach to introduce these variants into the endogenous mouse *Cfap410* gene instead of overexpressing these, so as to have a physiologically relevant model which recapitulates the effects seen in human ALS.

Herein, we have modified the coding sequence of the endogenous *Cfap410* gene in mouse ES cells to include the hemagglutinin (HA) epitope tag at the 3′ end of the protein. Subsequently, we have introduced mutations into the coding sequence at positions homologous to two mutations which have been identified previously in human cohorts,^6^ and we have previously predicted to have functional consequences.^25^ We have found that the ES cell derived motor neurons carrying either mutation are more susceptible to DNA damage and have an impaired DNA damage response.

## Results

### *Cfap410* transcript 1 is the major protein coding transcript in the developing brain and differentiating motor neurons

Ensembl shows eight mouse *Cfap410* transcripts (Ensembl: 1810043G02Rik) with two protein coding variants: 1810043G02Rik-201 encoding the 249aa isoform (Uniprot Q8C6G1) and Ensembl: 1810043G02Rik-202 encoding the 212aa isoform (Uniprot Q3U699). The longer of these two transcripts (Rik-201, 249aa) has the highest homology with the canonical human isoforms (Ensembl CFAP410-201 and 202, Uniprot O43822-1 and −3, Figure S1A). The N-terminal sequence is highly conserved with the positions and identities of the V20 and R73 residues we aimed to edit identical between mouse and human (Figure S1B).

To ensure the most relevant transcript was tagged with the HA epitope, we first studied the expression of the different *Cfap410* transcripts in a developmental series of mouse brain tissue and in motor neurons differentiated *in vitro* from P19 embryonal carcinoma cells. The expression of transcript 1 (Rik-201) increases only slightly during early embryonic brain development between E12.5 and E16.5. A more dramatic increase is seen from E16.5 to E18.5 which then continues postnatally to P0 and P15 in the cerebrum with a 3-fold increase over E12.5 expression levels (Figure S1C). This is not observed in the cerebellum, though there is still a gradual increase in expression up to P15. A similar trend is seen after E18.5 for transcript two (Rik-202) in the cerebrum, though at lower absolute levels (Figure S1D).

*Cfap410* transcript 1 expression also shows a similar expression pattern in differentiating P19 cells (Figure S1E). First, there is a gradual increase in expression up to the midpoint of the differentiation, then a more rapid increase to levels 2-fold higher than those seen in undifferentiated P19 cells. Little change is observed in the case of transcript two in differentiating P19 cells (Figure S1F). These data suggest transcript 1 and therefore the 249aa isoform of Cfap410 is the dominant isoform in the developing brain and differentiating motor neurons. Therefore, we chose to introduce the HA epitope tag into this isoform.

Knock-in of an HA epitope tag and point mutations into the *Cfap410* gene in mESCs.

**Figure 1:**
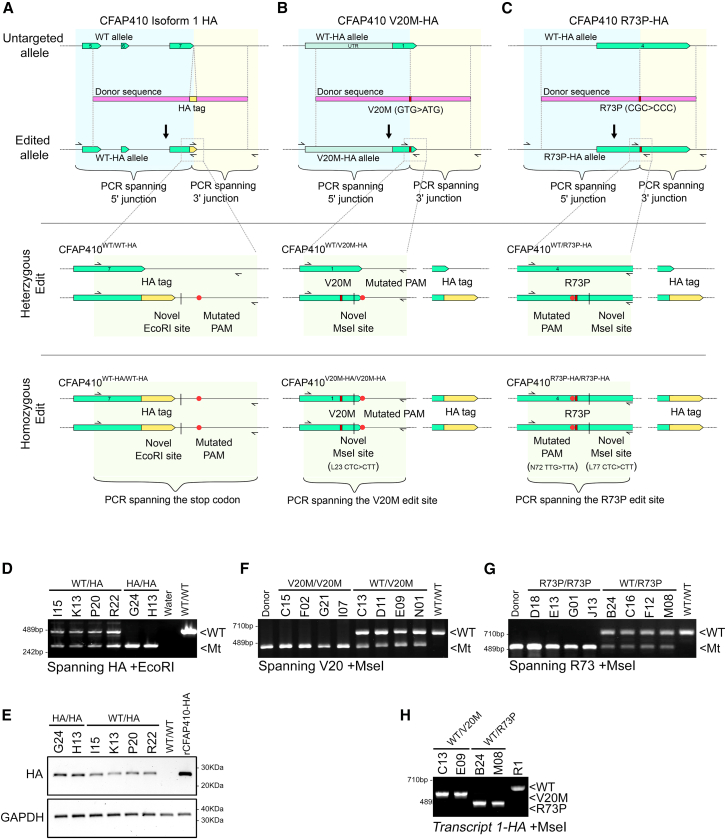
Sequential CRISPR/Cas9 gene editing the CFAP410 locus to introduce an HA tag and point mutations (A) Nickase Cas9 and paired guide sgRNAs targeting the stop codon of *CFAP410* isoform 1 (WT allele, green) were used to mediate the introduction of an HA tag (yellow) prior to the stop codon from a donor plasmid (Donor sequence, pink). The donor plasmid also carried a mutated PAM site for the 3′ guide (red dot) and novel EcoRI site (dash) used to screen for zygosity of the edit (see diagram detail in zoomed in section below comparing heterozygous and homozygous edits). Hetero- and homozygous HA-tagged clones were subsequently identified, and V20M or R73P point mutation (B and C) was then edited into the HA tagged allele to generate endogenously tagged variants. The donor plasmids carrying the V20M or R73P mutations also introduced synonymous base changes to leucine codons to create a novel MseI site, and mutated either the 3’ (V20M) or 5’ (R73P) PAM. (D) HA tag zygosity was determined by PCR across the locus followed by EcoRI digest (small arrows in A-C). (E) Western blot to show *CFAP410*-HA expression, immunoblotted using anti-HA antibody. GAPDH as loading control and transgenic R1 overexpressing HA tagged human *CFAP410* used as a positive control (R1 hC21HA). V20M and R73P zygosity was determined by PCR across the locus followed by MseI digest (F and G). Donor plasmid and R1 ESC parent DNA were used as mutant (Mt) and wildtype (WT) controls. (H) To determine whether heterozygous mutations were present in the HA tagged allele, RT-PCR was performed on HA-primed cDNA followed by MseI digest. Successful digestion indicated the point mutations were present on HA tagged allele.

The *Cfap410* gene was edited in R1 mESCs to introduce the HA epitope tag in either one or both alleles (Figure 1A). This was followed by the introduction of point mutation resulting in residue changes homologous to those identified in humans: V20M and R73P (Figures 1B and 1C).

Four pairs of guides were designed to target nickase Cas9 (nCas9) to the positions at which edits were to be introduced into the *Cfap410* gene. Guides were first screened for efficiency in R1 mESCs by transient transfection and indel frequency analysis by T7E1 assay.^27^ Guide pairs with the highest efficiency were then used to generate the indicated stable *Cfap410* variants: “HA tag” sgRNAs 18-11, “V20M” sgRNAs 5-2, and “R73P” sgRNAs 6–7, (Figures S2A–S2C; Table S2).

The donor constructs were introduced by electroporation into R1 mESC and clonal colonies isolated after enrichment through puromycin selection and magnetic sorting. We first introduced the HA tag prior to the stop codon in the final coding exon of transcript 1 of *Cfap410* (Figure 1A). Clones were screened by PCR for the successful introduction of the HA tag using a primer specific to the HA tag and a second to the endogenous *Cfap410* gene. Zygosity was determined using a novel EcoRI site introduced by the donor outside of the coding sequence (Figure 1A). The digestion of a fragment amplified by PCR across the stop codon results in shorter fragment where the HA tag is present, while the unedited product remains undigested. Two clones appeared to be homozygous for the HA tag while the remaining four appear to be heterozygous (Figure 1D). This was also confirmed directly by Sanger sequencing in homozygous clones (Figures S2D–S2E).

In heterozygous clones, the integrity of the wild type allele was determined by sequencing EcoRI resistant PCR products subcloned into pBluescript. Heterozygous clones with indels in the wild type allele were excluded from subsequent experiments. These clones were expanded and characterised further by PCR to amplify products that span from the HA tag to endogenous sequence (i.e., outside of the homology arms present in the donor plasmid) indicate the tag is present in the intended genomic site and not randomly inserted elsewhere. The expression of the HA tagged Cfap410 isoform was confirmed by western blot, where a band at 29 kDa is observed (Figure 1E).

Point mutations were then introduced into R1 *Cfap410* HA tagged clones, which were either homo- or heterozygous for the HA tag (i.e., *CFAP410*^HA/HA^ or *CFAP410*^+/HA^) in order to generate cell lines carrying heterozygous or homozygous HA tagged variants. The donor constructs for generating the V20M and R73P mutations were introduced by electroporation as before, and clones were first screened by melt curve analysis. The introduction of the point mutation, synonymous MseI site, and PAM mutation resulted in characteristic changes in the melt profile of PCR fragments spanning the edit site, which was confirmed by digestion with MseI.

These clones were expanded further and characterised for the nature of the edit or mutations that were present. PCR and Gel analysis of the clones identified by melt-curve analysis identified homozygous V20M or R73P mutations in transfected subclones of *Cfap410*^HA/HA^ R1 mESCs (Figures 1F and 1G), where the complete digestion of the PCR amplicon by MseI occurred (i.e., *Cfap410*^V20M-HA/V20M-HA^ or *Cfap410*^R73P-HA/R73P-HA^). Similarly, a correctly sized wild-type allele band and an MseI digested bands were used to further screen for heterozygous V20M or R73P mutations in transfected subclones of *Cfap410*^+/HA^ R1 mESCs. The presence of the mutations were again verified by Sanger sequencing, showing both the intended mutation and an intact wild-type allele in heterozygous clones (Figures S2F and S2G).

Heterozygous point mutations could have occurred in either the wild-type or HA tagged allele of *Cfap410*^+/HA^ cells. To determine which allele carried the mutation, cDNA from prospective clones was subjected to PCR using the *Cfap410* transcript 1 forward primer combined with an HA tag reverse primer and followed by digestion with MseI. PCR products from heterozygous clones produced digested products only when the novel MseI site was present in the amplicon i.e., the point mutation was present in the HA tagged allele (i.e., *Cfap410*^+/V20M-HA^ or *Cfap410*^+/R73P-HA^ (Figure 1H).

To summarise, throughout this manuscript the genotypes of these cell lines and genotypes will be referred by the edit made and its zygosity: *Cfap410*^*WT/WT*^ (unedited wild type cells), *Cfap410*^*HA/HA*^ (homozygous HA tagged wild type), *Cfap410*^*WT/V20M-HA*^ (heterozygous HA tagged V20M variant cells), *Cfap410*^*V20M-HA/V20M-HA*^ (homozygous HA tagged V20M variant cells), *Cfap410*^*WT/R73P-HA HA*^ (heterozygous HA tagged R73P variant cells), and *Cfap410*^*R73P-HA/R73P-HA*^ (homozygous HA tagged R73P variant cells). These genotypes and the two independent clones of each taken forward for further analysis are further summarized in Table 1.

**Table 1:** Summary of CRISPR edited mESC lines used in this manuscript

Identifier	Genotype	Clone IDs
CFAP410^WT/WT^	Unmodified original ES cell line	R1 mESC
CFAP410^WT/HA^	Heterozygous HA tag Knock-in subclone of R1 (second allele remains WT)	I15
CFAP410^HA/HA^	Homozygous HA tag Knock-in subclone of R1	G24 & H13
*CFAP410*^WT/V20M-HA^	Heterozygous V20M-HA tag, Subclone of I15 (second allele remains WT)	C13 & E09
*CFAP410*^V20M-HA/V20M-HA^	Homozygous HA tag Subclone of H13	C15 & F02
*CFAP410*^WT/R73P-HA^	Heterozygous R73P-HA tag, Subclone of I15 (second allele remains WT)	B24 & M04
*CFAP410*^R73P-HA/R73P-HA^	Homozygous R73P-HA tag, Subclone of H13	D14 & J13

### CFAP410 variant proteins are stable and associate with pericentrin

**Figure 2:**
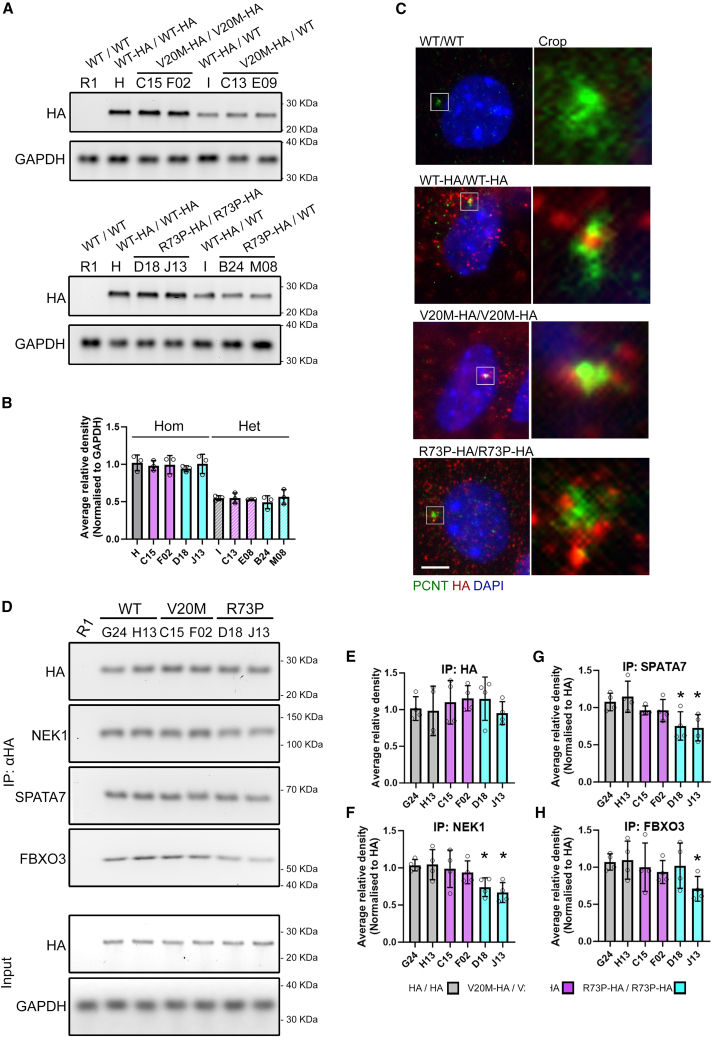
Expression of HA tagged CFAP410 variants (A) Western blot of two representative clones from each genotype immunoblotted using anti-HA antibody. (B) Quantified expression levels of CFAP410-HA normalized to GAPDH loading control from three replicate western blots (Data are represented as mean ± SEM). (C) Immunostaining for PCNT and HA in representative clones counterstained with DAPI. Inset shows a close crop of centriole and basal body. Scale bar 5 μm. Equivalent images for the heterozygous clones can be found in Figure S2. (D) Co-immunoprecipitation using an antibody against the HA tag and immunoblotting for NEK1, SPATA7 and FBXO3. Performed on neuronal culture lysates of R1 (*CFAP410*^WT/WT^), G24 and H13 (*CFAP410*^WT-HA/WT-HA^), C15 and F02 (*CFAP410*^V20M-HA/V20M-HA^), D18 and J13 (*CFAP410*^R73P-HA/R73P-HA^). Quantification of immunoprecipitated HA tagged *CFAP410* (E) and co-immunoprecipitated NEK1 (F), SPATA7 (G), and FBXO3 (H) from three replicate western blots (Data are represented as mean ± SEM). NEK1, SPATA7, and FBXO3 normalized to HA. Data compared by ANOVA with Bonferroni’s *post hoc* test. ∗*p* < 0.05.

Knocking in the point mutations R73P and V20M into the *Cfap410* gene in undifferentiated mESCs did not appear to affect the stability or levels of the HA tagged Cfap410 protein under normal culture conditions (Figures 2A and 2B). Levels of Cfap410 variant proteins in homozygous *Cfap410*^V20M/V20M^ or *Cfap410*^R73P/R73P^ cell lines were comparable to that seen in the parent cell line R1*Cfap410*^HA/HA^. Clones heterozygous for either V20M or R73P mutation showed protein levels approximately half the levels of Cfap410 seen in the homozygous clones (Figures 2A and 2B).

Immunostaining for the HA tag revealed staining in small puncta found primarily throughout the cytoplasm but also within the nucleus (Figure 2C). Co-staining with the centrosomal marker pericentrin (PCNT) identified an association of larger punctae adjacent to the centrosome (Figure 2C, insets). No gross differences in the distribution of the HA tag staining or association with PCNT were observed between wildtype *Cfap410*^WT/HA^ and *Cfap410*^HA/HA^ ESCs and the ESCs carrying the knock-in HA tagged variants (Figures 2C and S2H).

### *CFAP410* variants do not affect the frequency of primary cilia

Previous reports have suggested that CFAP410 is involved in the formation or maintenance of the primary cilia, and knockdown of CFAP410 reduced primary cilia formation in both mouse and human cell lines.^7^^,^^9^ To examine whether primary cilia were affected in cells with the knock-in *Cfap410* variants, ciliogenesis was induced in ESCs and NPCs either through serum starvation or the removal of EGF and FGF2, respectively (Figures S3A and S2B). Primary cilia frequency within the total population of cells was determined by staining with anti ARL13B antibody. ARL13B is a small GTPase that localizes to the ciliary axoneme.^28^ No significant differences were found in the frequency of cells with primary cilia between the control and *Cfap410* variant knock-in ESCs or NPCs (Figures S3D and S3E).

Variant cell lines were also differentiated to neuronal populations with dorsal spinal identities, containing ∼40% motor neurons. In these motor neuron enriched cultures, few ISL1/2 positive motor neurons possessed primary cilia in comparison to the total population (Figure S3C). Again, no significant difference in the frequency of primary cilia was observed between neurons/motor neurons derived from *Cfap10*^WT/WT^ and *Cfap410*^HA/HA^ clones and those carrying hetero- or homozygous^V20M-HA^ or ^R73P-HA^ alleles (Figure S3F). Furthermore, no clear differences in length or morphology of primary cilia were observed by staining the axoneme with an antibody to ARL13B. We also did not observe robust co-localization between the mitochondrial marker MitoTracker and wild-type HA tagged *Cfap410* or any of the variants in neurons and their neurites, where HA-tagged *Cfap410* is distributed throughout the neurite and cell body (Figure S4). A similar broad intracellular distribution has also been demonstrated by overexpression in HeLa.^22^ Although the gross structure of the primary cilia appears unaffected, further interrogation of the composition and arrangement of the ciliary complexes is required to assess the consequences of these mutations. Because of this, we sought to identify whether known CFAP410 interactions were impaired.

### Known protein-protein interactions are disrupted by Cfap*410* mutations

Although association with the basal body and overall frequency of primary cilia are unaffected by these Cfap410 variants, we found altered protein-protein interactions in motor neuron cultures with previously described factors^7^^,^^22^^,^^23^ - NEK1, SPATA7 and FBXO3 (Figure 2D). The anti HA tag antibody was used to immunoprecipitate WT-HA, V20M-HA, and R73P-HA Cfap410 proteins from motor neuron cultures differentiated from the parental R1 *Cfap410*^WT/WT^, *Cfap410*^HA/HA^, *Cfap410*^V20M-HA/V20M-HA^, and *Cfap410*^R73P-HA/R73P-HA^ ESCs. Co-immunoprecipitation confirmed these interactions occurred in the *Cfap410* ALS variant knock-in cells regardless of genotype (Figures 2E–2H). However, quantitation by densitometry indicated reduced interactions between *Cfap410*^R73P-HA^ and NEK1, SPATA7 in two independent clones tested, while interaction with FBXO3 was significantly reduced in only one *Cfap410*^R73P-HA^ line (Figures 2E–2H, *p* < 0.05).

### *Cfap410* variants do not affect ESC or NPC growth rates

Proteins associated with primary cilia often play a role in cell division and proliferation. Although primary cilia frequency and morphology appear unaltered, defects may still be present in cilia-associated processes. We first examined whether ALS-associated mutations in *Cfap410* would affect the rate of proliferation in ES cells as well as neural progenitor cells (NPCs) derived from these ES cells.

**Figure 3:**
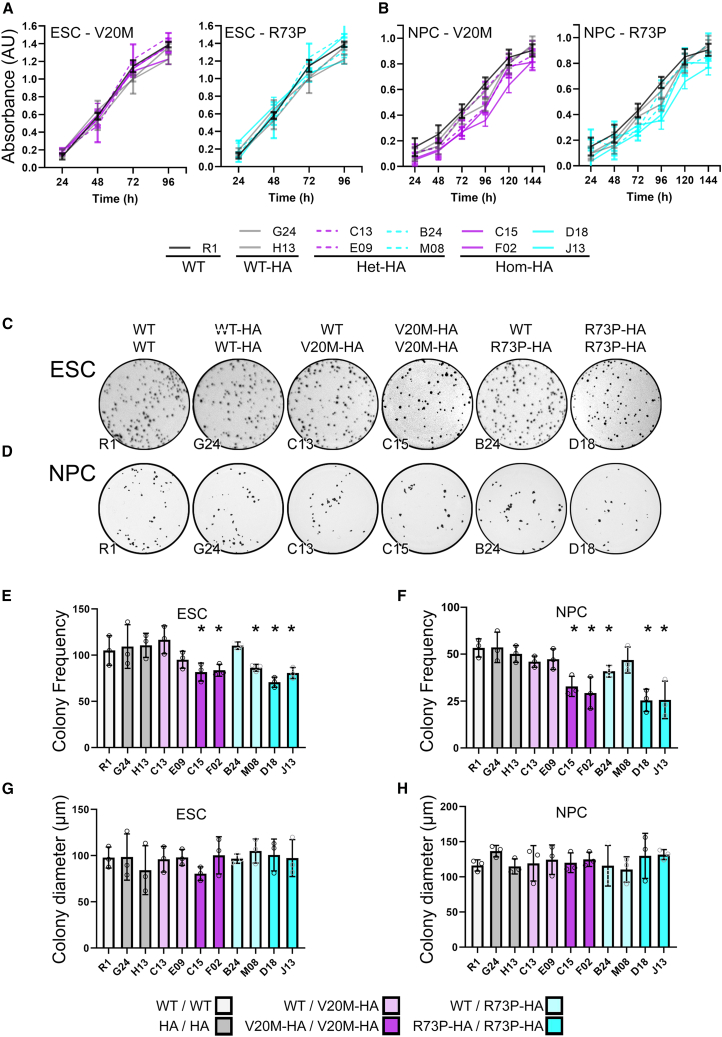
CFAP410 variants do not affect the proliferation and growth of ESC or NPC, but do affect clonal survival Proliferation was assayed over the indicated time courses with 24h intervals. Two clones of each ESCs (A) or NPCs (B) genotype were assayed alongside wildtype (WT) R1. Three experimental replicates were performed with triplicate assay wells in each. Data presented as mean absorbance ±SEM. Colony formation assays were also performed in triplicate with three replicates each of ESCs (C) and NPCs (D) of each genotype alongside R1 and stained with crystal violet. ESC and NPC colony frequency (E and F) was determined from each experiment and presented as mean frequency ±SEM. Data compared by ANOVA with Bonferroni’s *post hoc* test. ∗*p* < 0.05. ESC and NPC colony diameter (G and H) was determined from each experiment and presented as mean frequency ±SEM. Key to genotypes: WT, *CFAP410*^WT/WT^ (R1); WT-HA, *CFAP410*^WT-HA/WT-HA^ (G24 and H13); Het-HA, *CFAP410*^WT/V20M-HA^ (C13 and E09) and *CFAP410*^WT/R73P-HA^ (B24 and M08); Hom-HA, *CFAP410*^V20M-HA/V20M-HA^ (C15 and F02) and *CFAP410*^R73P-HA/R73P-HA^ (D18 and J13).

Proliferation was assayed by measuring the viable cell population using an MTT based assay at 24h intervals after seeding each cell line at equal densities. At each interval during the four-day period during which ES cell proliferation was assayed, both R1 *Cfap410*^WT/WT^ and *Cfap410*^HA/HA^ cell lines showed comparable cell density, with some plateauing in growth rate after 72h (Figure 3A). In contrast, the heterozygous *Cfap410*^WT/V20M-HA^ and *Cfap410*^WT/R73P-HA^ cell lines showed a trend of reduced cell density at comparable time points (Figure 3A), as do the homozygous *Cfap410*^V20M-HA/V20M-HA^ and *Cfap410*^R73P-HA/R73P-HA^ lines.

Despite this, no significant difference between the rates of proliferation was observed between any cell line (0.553–0.677 ΔA/24h by MTT between days 1 and 3) and differences in cell density were only observable after 24h (Figure 3A), suggesting reduced survival after initial seeding may account for the overall lower cell density throughout the experiment.

ES cells of each genotype were differentiated to NPC identity, expanded, and characterized to ensure comparisons were being made between equivalent cell populations. Regardless of genotype, qRT-PCR showed no significant difference in the expression of the NPC markers *Nestin*, *Pax6* and *Sox2* (Figure S5A). In addition, each clone expressed the intermediate filament nestin in a characteristic NPC-like distribution (Figure S5B).

The growth characteristics of the NPCs followed a pattern similar to those observed for ES cells (Figure 3B). Over the six day time course, the density of *Cfap410*^WT/WT^ and *Cfap410*^HA/HA^ NPCs was consistently above those of *Cfap410*^WT/V20M-HA^ and *Cfap410*^WT/R73P-HA^ NPCs. Homozygous *Cfap410*^V20M-HA/V20M-HA^ and *Cfap410*^R73P-HA/R73P-HA^ NPCs had the lowest densities at each time point. Despite this, the proliferation rates were not significantly different (0.171–0.221 ΔA/24h by MTT between days 2 and 5, Figure 3B), and again the differences in cell density appear to stem from the earliest time point.

Colony formation assays were performed for both ESC and NPC to further investigate survival and growth characteristics. The frequency of colonies formed negatively correlated with zygosity (Figures 3C and 3D). The downward trend in colony frequency observed for the two *Cfap410*^WT/V20M-HA^ ESC lines was not significantly different from that of the *Cfap410*^WT/WT^ and *Cfap410*^HA/HA^ ESCs (Figure 3E). The reduction in colony frequency for the *Cfap410*^WT/R73P-HA^ ESC lines assayed was only significant in the case of one of the two lines (M08, *p* < 0.05). In contrast, the colony frequencies were halved where the ES cells were homozygous for both the *Cfap410*^V20M-HA^ and *Cfap410*^R73P-HA^ encoding alleles (*p* < 0.05, Figure 3E). Similar results were observed for colony frequency in assays performed with the equivalent genotypes of NPCs (Figure 3F). Despite these differences in colony frequency, no significant differences were found between the median diameters of the colonies that did arise during the assays for with ESCs or NPCs of any genotype (median ESC colony diameter 122.2 μm, σ 18.1 μm; Median NPC colony diameter 95.9 μm, σ 7.1 μm) (Figures 3G and 3H).

This corresponds with the previous data showing no difference in proliferation rates between the different genotypes in both ESC and NPCs (Figures 3A and 3B). These data suggest that the lag in growth observed during the early stages of the proliferation assays is likely due to increased cell death post-plating, reducing the initial population size, potentially due to increased sensitivity to stress during passaging and plating at low starting densities.

### ESCs, NPCs, and neurons carrying *Cfap410* variant knock-ins are more sensitive to stress in a zygosity-dependent manner

**Figure 4:**
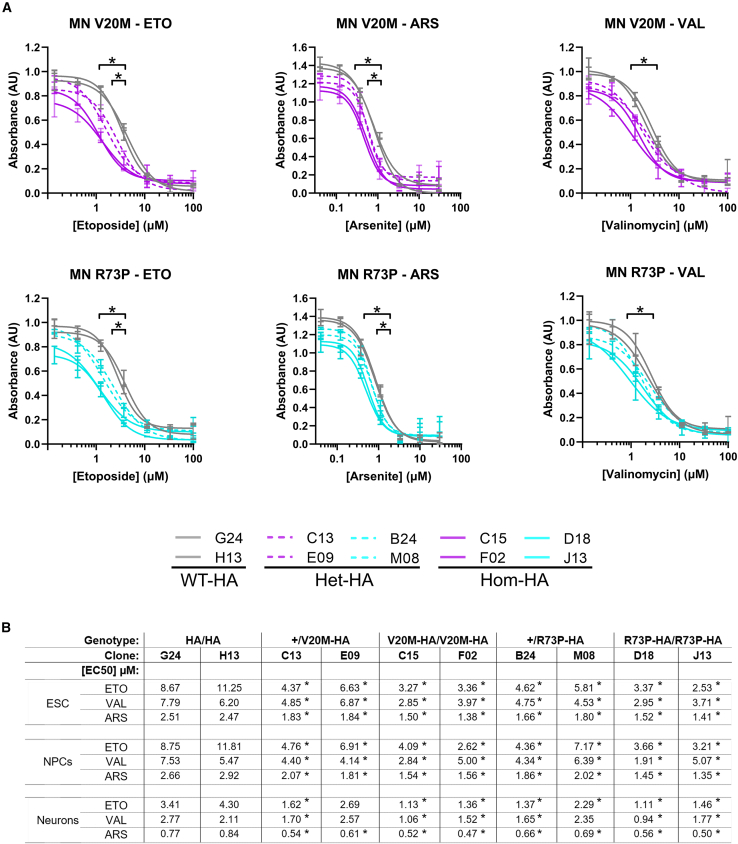
CFAP410 variants increase vulnerability to stress in differentiated neuronal cultures (A) Kill curves of etoposide (ETO), valinomycin (VAL) or sodium arsenite (ARS) treated motor-neuron directed differentiation of *CFAP410* variants ESCs. Viability determined after 24h. Three experimental replicates were performed with triplicate assay wells in each. Data presented as mean absorbance ±SEM. All X axes are on a log scale. (B) Summary table of EC50 concentrations for neurons, ESCs, and NPCs determined from a 4-parameter nonlinear dose–response fitted curve. ∗*p* < 0.05. Full response curves for ESC and NPCs can be found in Figure S6. Concentrations of Drugs: Etoposide and Valinomycin 100.00, 33.33, 11.11, 3.70, 1.23, 0.41, 0.14 and 0.00 μM, Sodium arsenite 30.00, 10.00, 3.33, 1.11, 0.37, 0.12, 0.04, 0.00 μM. Key to genotypes: WT, *CFAP410*^WT/WT^ (R1); WT-HA, *CFAP410*^WT-HA/WT-HA^ (G24 and H13); Het-HA, *CFAP410*^WT/V20M-HA^ (C13 and E09) and *CFAP410*^WT/R73P-HA^ (B24 and M08); Hom-HA, *CFAP410*^V20M-HA/V20M-HA^ (C15 and F02) and *CFAP410*^R73P-HA/R73P-HA^ (D18 and J13).

Sensitivity to stress was assayed in neuronal populations differentiated to dorsal spinal identities, containing ∼40% motor neurons (Figure 4), as well as in ESCs and NPCs (Figure S6).

In order to assay differences in the sensitivity of cells to stress, we generated dose-response curves for three different stressors: the ROS-generating oxidative stressor sodium arsenite (ARS),^29^ the topoisomerase II inhibitor Etoposide (ETO), which promotes DNA double-strand breaks^30^; and mitochondrial stressor valinomycin, which uncouples K^+^ transport across membranes.^31^ ESCs, NPCs, and neurons were subjected to varying concentrations of each stressor, and cell viability was determined 24 h later by MTT assay (Figures S5 and S6).

Cultures of motor neurons differentiated from *Cfap410* variant ESCs were consistently more vulnerable to each stressor (a lower [EC50]) than the equivalent from wild-type ESCs (Figure 4). In the case of each drug, a lower [EC50] was observed where the variant was present homozygously in comparison to heterozygotes. While the mean [EC50] for the wild-type HA tagged clones was 3.85, 2.44, and 0.81 μM of ETO, VAL, and ARS, respectively, lower mean [EC50] were observed for the heterozygous *Cfap410*^WT/V20M−HA^ clones (ETO: 2.15 μM, VAL: 2.13 μM, ARS: 0.58 μM) and *Cfap410*^WT/R73P-HA^ clones (ETO: 1.83 μM, VAL: 2.00 μM, ARS: 0.68 μM). [EC50] were lower still in the case of *Cfap410*^V20M-HA/V20M-HA^ clones (ETO: 1.24 μM, VAL: 1.29 μM, ARS: 0.51 μM) and *Cfap410*^R73P-HA/R73P-HA^ clones (ETO: 1.28 μM, VAL: 1.35 μM, ARS: 0.53 μM). We did not observe any difference between the two different variants (V20M or R73P) to the effects of the drug in these assays. The same trends were observed in both *Cfap410* variant ESCs and NPCs ([EC50] values are summarised in Figure 4B, full kill curves can be found in Figure S6).

### Motor neurons carrying *Cfap410* variants are more vulnerable to stress

**Figure 5:**
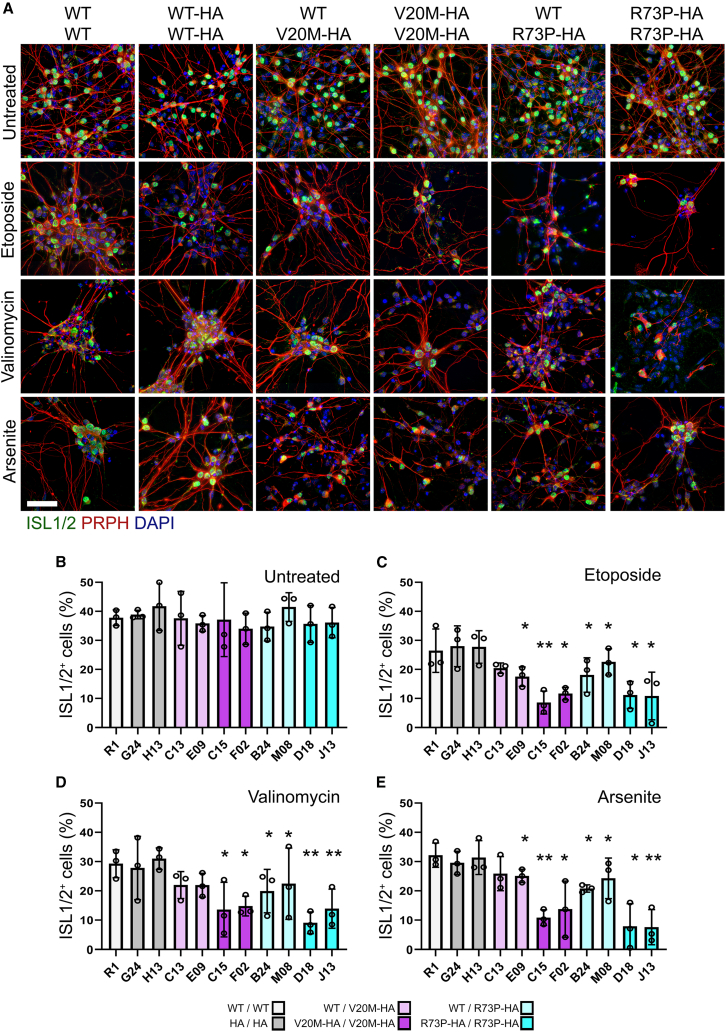
CFAP410 variant motor neurons are more vulnerable to genotoxic stress (A) Immunostaining for the motor neuron markers islet1/2 and peripherin (ISL1/2, PRPH) in neuronal cultures differentiated from *CFAP410* variant ESC clones and treated with etoposide, valinomycin, or sodium arsenite for 24h. Representative images from a single clone of each genotype are shown. Scale bar 100 μm. Average median Islet positive nuclei frequency was quantified from four random fields, each from three experiments (Bars represent data as mean ± SEM, data points represent experimental medians) (B) without treatment, or in response to (C) etoposide, (D) valinomycin, or (E) sodium arsenite. Data compared by ANOVA with Bonferroni’s *post hoc* test. ∗*p* < 0.05, ∗∗*p* < 0.005. Key to genotypes: WT/WT, *CFAP410*^WT/WT^ (R1); HA/HA, *CFAP410*^WT-HA/WT-HA^ (G24 and H13); WT/V20M-HA, *CFAP410*^WT/V20M-HA^ (C13 and E09); WT/R73P-HA, *CFAP410*^WT/R73P-HA^ (B24 and M08); V20M-HA/V20M-HA, *CFAP410*^V20M-HA/V20M-HA^ (C15 and F02); R73P-HA/R73P-HA, *CFAP410*^R73P-HA/R73P-HA^ (D18 and J13).

The differentiation protocol we used directs ESCs toward motor neuron identity; however, the resulting neuronal populations are not pure (Figure 5A). Within the differentiated cultures, motor neurons were identified by their expression of the transcription factors islet-1/2 (ISL), and the intermediate filament peripherin (PRPH).^32^ We did not find any significant differences in the frequency of ISL^+^ neurons between *Cfap410*^WT/WT^ or *Cfap410*^HA/HA^ cultures and the hetero- or homozygous *Cfap410* ALS variant carrying neurons under normal culture condition (Figure 5B).

To investigate the effects of stress on motor neuron survival, cultures were treated with ETO, VAL or ARS at concentrations equivalent to the respective mean IC50 values determined for *Cfap410* variant cultures. In all cases, motor neurons were more vulnerable than the total neuronal population, as fewer motor neurons survive after drug treatment. Motor neurons homozygous for *Cfap410* ALS variants were more susceptible to drug treatment. ETO, VAL, and ARS treatment reduced the mean motor neuron frequencies of the control *Cfap410*^WT/WT^ or *Cfap410*^HA/HA^ cultures to 29% (σ 4.8%), 27% (σ 7.5%), and 30% (σ 4.2%), respectively (Figure 5B). ETO treatment significantly reduced the frequency of motor neurons to below 15% in *Cfap410*^V20M-HA/V20M-HA^ (Clone C15 $\bar{x}$ 13.6%, σ 9.3%; Clone F02 $\bar{x}$ 14.8%, σ 3.4%) and *Cfap410*^R73P-HA/R73P-HA^ (Clone D14 $\bar{x}$ 9.1%, σ 3.7%; Clone J13 $\bar{x}$ 13.9%, σ 6.7%) (Figure 5C, *p* < 0.005). Similar trends were seen with VAL and ARS treatments. With VAL treatment, the mean frequency of surviving motor neurons in the case of *Cfap410*^V20M-HA/V20M-HA^ cultures was 8.6% (C15, σ 4.0%) and 11.7% (F02, σ 2.2%). In the case of *Cfap410*^R73P-HA/R73P-HA^ cultures, the mean frequency of surviving motor neurons was 11.2% (D14, σ 4.7%) and 10.9% (J13, σ 8.2%) (Figure 5D, *p* < 0.005). With ARS treatment, the mean surviving motor neuron frequencies in *Cfap410*^V20M-HA/V20M-HA^ cultures was 10.9% (C15, σ 2.7%) and 13.8% (F02, σ 9.6%) and in the case of *Cfap410*^R73P-HA/R73P-HA^ cultures was 7.9% (D14, σ 4.7%) and 7.6% (J13, σ 4.0%) (Figure 5E, *p* < 0.005).

Surviving motor neuron frequency was also reduced in heterozygote cultures compared to wildtype, but not to the same extent as in homozygote cultures. ETO treatment reduced the mean motor neuron frequency in *Cfap410*^WT/V20M-HA^ cultures to 22.0% (C13, σ 4.8%) and 21.2% (E09, σ 4.9%), and in *Cfap410*^WT/R73P-HA^ cultures to 19.9% (B24, σ 7.4%) and 22.5% (M04, σ 12.2%) (Figure 5C, *p* < 0.05). VAL treatment reduced the mean motor neuron frequency in *Cfap410*^WT/V20M-HA^ cultures to 20.4% (C13, σ 1.8%) and 17.5% (E09, σ 4.2%), and in *Cfap410*^WT/R73P-HA^ cultures to 18.1% (B24, σ 5.9%) and 22.6% (M04, σ 4.6%) (Figure 5D, *p* < 0.05). Finally, ARS treatment reduced the mean motor neuron frequency in *Cfap410*^WT/V20M-HA^ cultures to 25.9% (C13, σ 5.8%) and 25.1% (E09, σ 2.3%), and in *Cfap410*^WT/R73P-HA^ cultures to 20.8% (B24, σ 1.3%) and 24.3% (M04, σ 6.9%) (Figure 5D, *p* < 0.05).

No significant differences were found between motor neuron frequency in *Cfap410*^WT/V20M-HA^ and *Cfap410*^WT/R73P-HA^ cultures treated with ETO or VAL. A higher frequency of motor neurons in *Cfap410*^WT/V20M-HA^ cultures survived relative to *Cfap410*^WT/WT^ or *Cfap410*^HA/HA^ cultures after ARS treatment in comparison to ETO or VAL. A similar trend is also observed in ARS treated homozygous *Cfap410*^V20M-HA/V20M-HA^ in comparison to the *Cfap410*^R73P-HA/R73P-HA^. This suggests that the V20M mutation has a less severe effect on Cfap410 function in response to certain types of stress.

**Figure 6:**
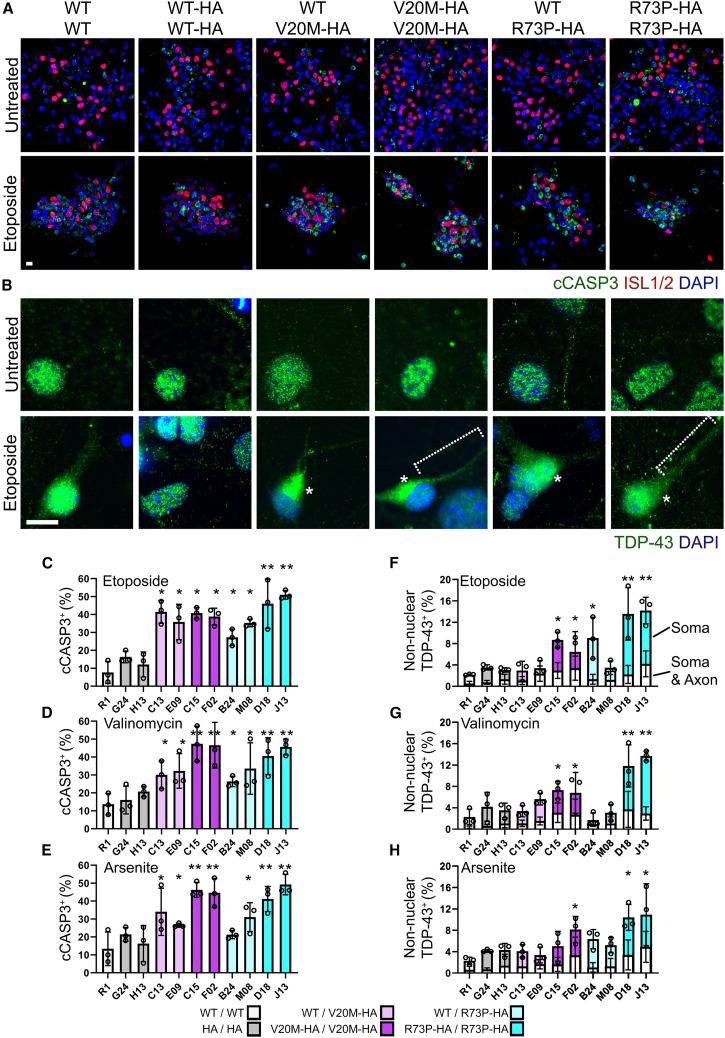
Increased apoptosis and TDP-43 redistribution in CFAP410 variant neurons (A and B) Immunostaining for (A) cleaved caspase 3 (cCASP3) and ISL1/2, or (B) TDP-43 in neurons differentiated from *CFAP410* variant ESCs and treated with etoposide for 24h. Representative images from a single clone of each genotype are shown. Scale bar 10 μm. Average median cleaved caspase 3 positive nuclei frequency was quantified from four random fields, each from three experiments (Bars represent data as mean ± SEM, data points represent experimental medians) in response to (C) etoposide, (D) valinomycin or (E) sodium arsenite. Data compared by ANOVA with Bonferroni’s *post hoc* test. ∗*p* < 0.05, ∗∗*p* < 0.005. Cells with TDP-43 staining in the soma (asterisk), or in the primary axon (dashed bracket) were quantified from four random fields each from three experiments (Bars represent data as mean ± SEM, data points represent experimental medians) in response to (F) etoposide, (G) valinomycin or (H) sodium arsenite. Data compared by ANOVA with Bonferroni’s *post hoc* test. ∗*p* < 0.05, ∗∗*p* < 0.005. Immunostaining after treatment with valinomycin or sodium arsenite, as well as quantification of baseline Caspase 3 cleavage in untreated cells, can be found in Figure S7. Key to genotypes: WT/WT, *CFAP410*^WT/WT^ (R1); HA/HA, *CFAP410*^WT-HA/WT-HA^ (G24 and H13); WT/V20M-HA, *CFAP410*^WT/V20M-HA^ (C13 and E09); WT/R73P-HA, *CFAP410*^WT/R73P-HA^ (B24 and M08); V20M-HA/V20M-HA, *CFAP410*^V20M-HA/V20M-HA^ (C15 and F02); R73P-HA/R73P-HA, *CFAP410*^R73P-HA/R73P-HA^ (D18 and J13).

During apoptosis, caspase-3 (CASP3) is cleaved and translocates to the nucleus.^33^ Immunostaining for cleaved caspase-3 (cCASP3) in neuronal cultures treated with drugs shows robust nuclear localization, with many cells also showing condensed fragmented DNA seen by DAPI staining (Figures 6 and S7).

Co-immunostaining for cCASP3 and ISL1/2 failed to show cells expressing both the motor neuron marker and nuclear cCASP3. This may be because vulnerable motor neurons have apoptosed and been lost prior to fixation and observation at this time point, or ISL1/2 may have already been degraded during apoptosis in the remaining cells positive for nuclear cCASP3. Despite this, the frequency of cCASP3 present in the total neuronal population after drug treatment correlates with the degree of motor neuron loss observed in Figure 5. Untreated neuronal cultures showed around 1–7% cCASP3 positive nuclei, with homozygous *Cfap410* variant neurons trending higher but not significantly so. After treatment with ETO, 5–15% of nuclei in *Cfap410*^WT/WT^ and ^HA/HA^ cultures are cCASP3^+^ while 3– to 4-fold more neuronal nuclei are cCASP3^+^ in both homo- and heterozygous *Cfap410* variant neuronal cultures (*p* < 0.05, Figure 5C), particularly in the case of both *Cfap410*^R73P-HA/R73P-HA^ clones (*p* < 0.005). Similar trends were seen after treatment with VAL and ARS, however, fewer cCASP3^+^ nuclei were observed in heterozygous *Cfap410* variant neurons when compared to *Cfap410* homozygous neurons as compared with ETO treatment. Neurons homozygous for the *Cfap410*^*V20M-*HA^ and *Cfap410*^*R73P-HA*^ ALS variants had an increased frequency of cCASP3^+^ nuclei.

The redistribution of nuclear TDP-43 to the cytoplasm is observed in nearly 97% of patients with ALS.^34^ In order to see if this happened in neurons with the knock-in Cfap410 variants, we stained for TDP-43 in control and *Cfap410* variant neurons. TDP-43 was observed at low frequency in the soma of control neurons after treatment with ETO (Figure 6B,F-H), VAL, and ARS (∼2–4%, Figure S7B). The frequency of neurons in which this was observed was higher in the heterozygous *Cfap410*^WT/V20M-HA^ and *Cfap410*^WT/R73P-HA^ neuronal cultures in comparison to control (∼6–9%, *p* < 0.05, Figures 6F–6H). The nuclear to cytoplasmic redistribution was more frequently observed in homozygous clones, particularly *Cfap410*^R73P-HA/R73P-HA^ (13.5–14.2%, σ 2.4–4.9%) as compared to *Cfap410*^V20M-HA/V20M-HA^ (8.7–6.4%, σ 1.5–3.8%). In a small number of cells, TDP43 was observed not only in the soma but also along the neurites (Figures 6F–6H, white bars). This was particularly noticeable in cultures of *Cfap410*^R73P-HA/R73P-HA^ neurons after ETO treatment, where 2.7% (σ 1.6%) of neurons showed TDP43 localised in the soma and neurites in comparison to 0.9% (σ 0.6%) of control neurons.

### *Cfap410* variant knock-in ESCs, NPCs, and neurons are more susceptible to DNA damage

Cfap410 has been implicated in the DNA damage response (DDR) and is seen to be associated with other DDR proteins such as NEK1.^22^ Since we observed reduced interactions between Cfap410 variants and Nek1 by co-IP, we examined how the DNA damage response was activated in response to stress upon treatment with ETO, VAL, and ARS. Histone H2AX is phosphorylated on Ser-139 (γH2AX) and forms foci where double strand DNA breaks have occurred.^35^ We quantified γH2AX levels in response to drug treatment in control and *Cfap410* variant knock-in ESCs, NPCs, and neuronal cultures to see the effects on the DNA damage response.

**Figure 7:**
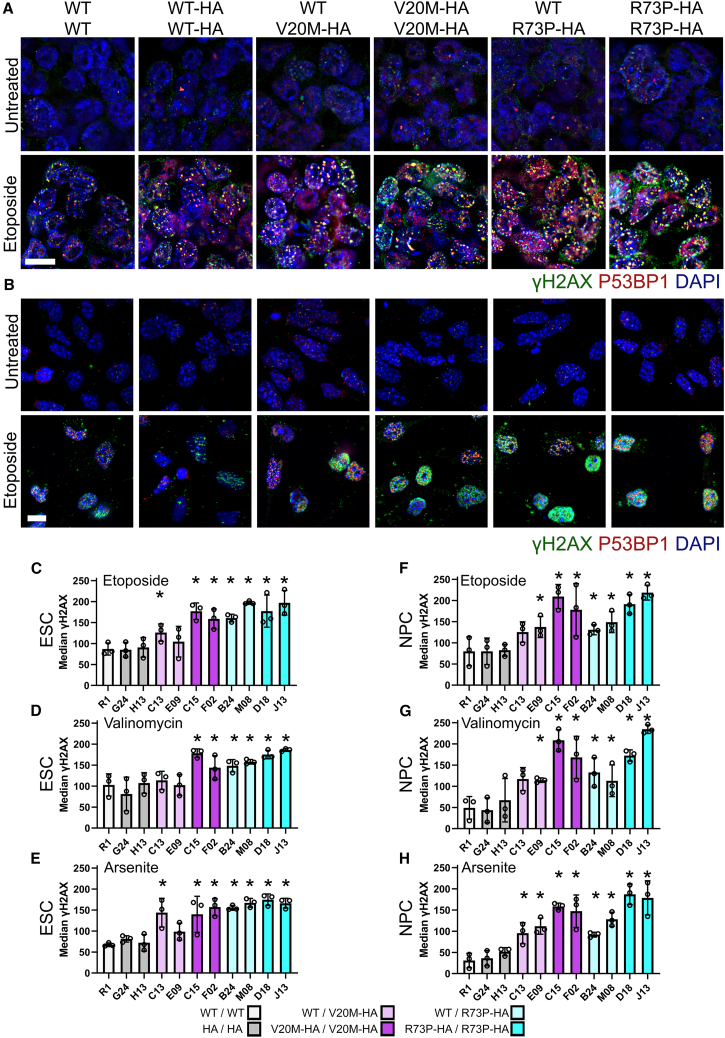
ESC and NPC lines carrying *CFAP410* variants show increased DNA damage response (A and B)Immunostaining for γH2AX and P53BP1 in CFAP410 variant ESCs (A) and NPCs (B) treated with etoposide for 24h. Representative images from a single clone of each genotype shown. Scale bar 10 μm. Average median nuclear γH2AX intensity in ESCs was quantified from four random fields each from three experiments (Bars represent data as median ±SEM, data points represent experimental medians) in response to (C) etoposide, (D) valinomycin, or (E) sodium arsenite. Average median nuclear γH2AX intensity in NPCs was quantified from four random fields each from three experiments (Bars represent data as mean ± SEM, data points represent experimental medians) in response to (F) etoposide, (G) valinomycin, or (H) sodium arsenite. Data compared by ANOVA with Bonferroni’s *post hoc* test. ∗*p* < 0.05. Immunostaining after treatment with valinomycin or sodium arsenite as well as the baseline quantification of γH2AX in untreated ESC and NPC can be found in Figure S8C and D. Key to genotypes: WT/WT, *CFAP410*^WT/WT^ (R1); HA/HA, *CFAP410*^WT-HA/WT-HA^ (G24 and H13); WT/V20M-HA, *CFAP410*^WT/V20M-HA^ (C13 and E09); WT/R73P-HA, *CFAP410*^WT/R73P-HA^ (B24 and M08); V20M-HA/V20M-HA, *CFAP410*^V20M-HA/V20M-HA^ (C15 and F02); R73P-HA/R73P-HA, *CFAP410*^R73P-HA/R73P-HA^ (D18 and J13).

Immunostaining for both γH2AX and P53BP1 showed a low level of DDR in ESCs (Figure 7A) and NPCs (Figure 7B). Quantification showed no significant differences between all the untreated cell lines (Figure S8C–S8D). The treatment of ESCs with ETO (Figure 7A), VAL or ARS (Figure S7A) resulted in increased levels of γH2AX and P53BP1 in the nucleus in a genotype dependent manner. Quantification consistently showed that significantly more γH2AX was present in the nucleus of ESCs carrying either the *Cfap410*^*V20M-HA/V20M-HA*^ or *Cfap410*^*R73P-HA/R73P-HA*^ ALS variants (*p* < 0.05, Figures 7C–7E). Of the heterozygous clones, only those with the genotype *Cfap410*^WT/R73P-HA^ showed significantly increased γH2AX levels in comparison to *Cfap410*^WT/WT^ and ^HA/HA^. In the case of *Cfap410*^WT/V20M-HA^, only clone C13 was significantly different from the controls after ETO or ARS treatment, but not upon treatment with VAL (*p* < 0.05, Figures 7C–7E).

A similar trend was observed in ETO, VAL or ARS treated NPCs (Figures 7F–7H). However, here the *Cfap410*^WT/R73P-HA^ clones showed a reduced but still significant response in comparison to the equivalent ESC clones (Figures 7C–7E). The heterozygous *Cfap410*^WT/R73P-HA^ clones were affected significantly when compared to the controls, unlike the *Cfap410*^WT/V20M-HA^ when treated with ETO and VAL.

**Figure 8:**
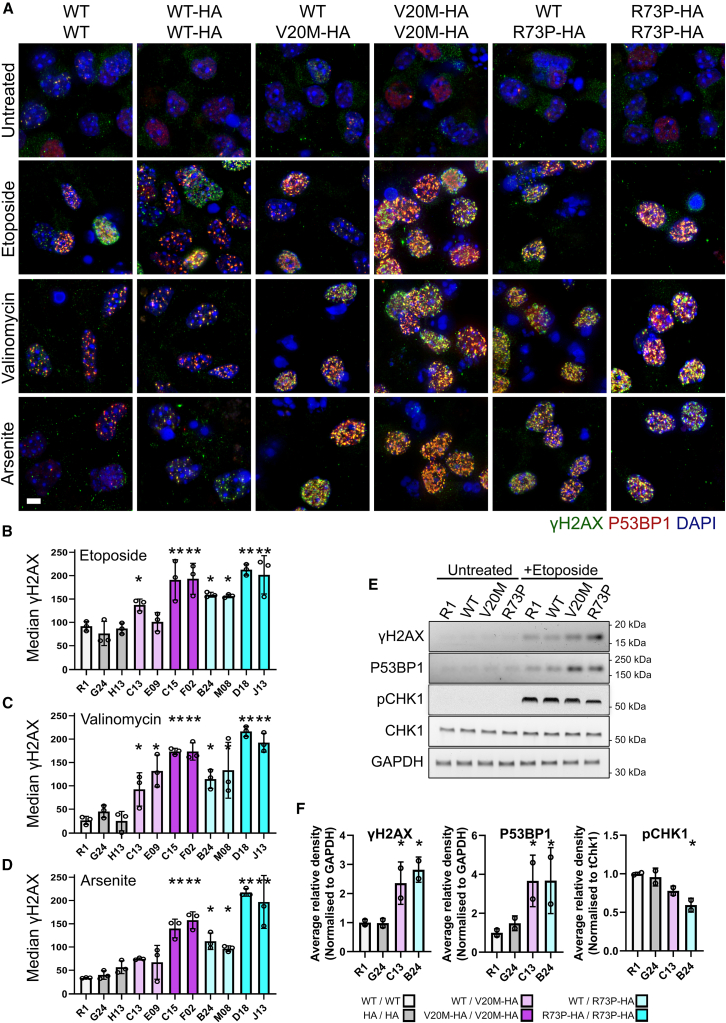
Neurons carrying CFAP410 variants show increased DNA damage response (A) Immunostaining for γH2AX and P53BP1 in neurons differentiated from CFAP*410* variant ESCs and treated with etoposide, valinomycin, or sodium arsenite for 24h. Representative images from a single clone of each genotype are shown. Scale bar 10 μm. Average median nuclear γH2AX intensity in neurons was quantified from four random fields each from three experiments (Bars represent data as mean ± SEM, data points represent experimental medians) in response to (B) etoposide, (C) valinomycin, or (D) sodium arsenite. Data compared by ANOVA with Bonferroni’s *post hoc* test. ∗*p* < 0.05, ∗∗*p* < 0.005. Baseline quantification of γH2AX in untreated MNs is shown in Figure S8E. (E) Representative Western blot of untreated and etoposide treated cells for γH2AX, P53BP1, phospho and total CHK1. GAPDH loading control. (F) Quantification of two replicate western blots for γH2AX, P53BP1 (normalized to GAPDH), and pCHK1 (normalized to total CHK1) (Bars represent data as mean ± SEM, data points represent experimental medians). Key to genotypes: WT/WT, *CFAP410*^WT/WT^ (R1); HA/HA, *CFAP410*^WT-HA/WT-HA^ (G24 and H13); WT/V20M-HA, *CFAP410*^WT/V20M-HA^ (C13 and E09); WT/R73P-HA, *CFAP410*^WT/R73P-HA^ (B24 and M08); V20M-HA/V20M-HA, *CFAP410*^V20M-HA/V20M-HA^ (C15 and F02); R73P-HA/R73P-HA, *CFAP410*^R73P-HA/R73P-HA^ (D18 and J13).

In untreated motor neuron cultures, minimal γH2AX levels were observed, as was the case with ESC and NPC cultures (Figure S8A, quantified in Figure S8E). A trend of increased γH2AX levels in untreated homozygous *Cfap410* ALS variant neurons was observed, but this did not appear to be significant (*p* > 0.05) (Figures 8A–S8E). After treatment with ETO, γH2AX and P53BP1 levels were highest in *Cfap410*^V20M-HA/V20M-HA^ and *Cfap410*^R73P-HA/R73P-HA^ neurons (Figure 8A). The quantification of this after ETO treatment showed that neuronal cultures differentiated from two independent clones homozygous for either of the *Cfap410* ALS variants have γH2AX levels significantly higher than controls (Figure 8B, *p* < 0.005). The same trends and significant differences in γH2AX levels were found in VAL (Figure 8C) and ARS treated neurons (Figure 8A, quantified in Figure 8D). With both VAL and ARS treatment, the responses observed in neurons differentiated from either sets of heterozygous clones (i.e., both clones of *Cfap410*^*WT/V20M-HA*^ and *Cfap410*^*WT/R73P-HA*^) were muted.

Western blots were performed to confirm the increased γH2AX and P53BP1 levels observed by immunofluorescence for ETO treated neurons (Figure 8E). Here, heterozygous clones were used to better represent the patient-associated genotype. Densitometry showed significantly higher amounts of γH2AX and P53BP1 present in *Cfap410* variant neurons after ETO treatment (Figure 8F). ETO is known to activate the ATR-CHK1 DDR pathway in neurons and other cell types so we examined whether the phosphorylation of CHK1 was also occurring here.^36^^,^^37^ Immunoblotting showed no changes in total CHK1 levels between WT and ALS variant *Cfap410* neurons (Figures 8E and 8F). Little to no phospho-CHK1 (pCHK1) could be detected in untreated neurons; however, levels were increased after ETO treatment in all cultures, though to a lesser degree in *Cfap410* ALS variant neurons (Figure 8F). When comparing between genotypes, the levels of phospho-CHK1 present in *Cfap410*^WT/V20M-HA^ and *Cfap410*^WT/R73P-HA^ appeared subdued. Though we may have observed a more dramatic effect in homozygous lines, we could not exclude the possibility that the gene product from the unedited allele could compensate for the mutation in the other. This does not appear to be the case, and we see a DDR phenotype in the heterozygous cells, suggesting the same could occur *in vivo*.

## Discussion

CFAP410 has been reported to be a component of the basal body of primary cilia, and mutations in this protein have been found to be associated with ciliopathies.^7^^,^^8^^,^^9^^,^^14^ Primary cilia have been shown to play an anti-apoptotic role in the central nervous system^38^ and defects in primary cilia could lead to the increased vulnerability of cells through multiple mechanisms such as inappropriate cell cycle re-entry, autophagy defects, or the inability to transduce protective signaling.^38^^,^^39^^,^^40^

CFAP410 has been shown to localize in ciliary structures of the photoreceptor cells.^9^ We found that in cells with HA tagged wild type and both the predicted functional variant knock-in cells we studied, HA tagged mouse Cfap410 localized to the basal body. However, we did not observe loss of cilia or any gross morphological effects on primary cilia in the neurons with the ALS variant knock-ins, and previous reports have shown either complete loss of cilia with lowered CFAP410 levels or only partial loss of cilia upon complete knock-out, suggesting a mutation or cell-type dependent effect.^10^^,^^14^ Wheway et al.,^7^ were able to partially rescue ciliogenesis in Cfap410 knock-down mouse kidney epithelial cells by expressing the R73P variant. Stadler et al.,^41^ have found that the C-terminal of CFAP410 is essential to its localization at the basal body, suggesting the mutations characterized here are too distal to interfere with this interaction. This highlights a key difference between loss of CFAP410 through knock-out or knock-down in comparison to point mutations.

Together, these indicate that although we find primary cilia are present, and that mutant Cfap410 variants continue to associate with the basal body, there are likely functional differences, as shown by the altered protein-protein interactions by immunoprecipitation. It is also possible that the two variants we knocked into the Cfap410 gene have caused subtle changes to the primary cilia composition and function. Further characterization of primary cilia, basal body components, and associated signaling pathways in the Cfap410 ALS variant knock in cells may identify more subtle defects if present.

Previous studies have demonstrated a role for CFAP410 and its interactor NEK1 in the DNA damage response, and an ALS associated variant in NEK1 shows increased DNA damage in motor neurons.^7^^,^^18^^,^^19^^,^^20^^,^^22^ We also found that ESCs, NPCs, and MNs carrying either the V20M or R73P variants were more susceptible to DNA damage when subjected to genotoxic stress, despite the V20M variant showing no significant differences in NEK1 interaction by IP. However, we found that heterozygous cells with the V20M variant were less susceptible than those with the R73P variant.

In the V20M *Cfap410* variant, the residue change is from a hydrophobic to a polar one, and in addition, there is a change from a shorter side chain to a longer one. Since V20 is conserved across species, we previously predicted that there is likely to be phenotypic consequences of this change^25^ which we do see in the form of a subtle increase in susceptibility to DNA damage.

The R73P variant of *Cfap410* has been implicated in a ciliopathy-Axial spondylometaphyseal dysplasia as reported by Wang et al.,.^8^ The effect of the R73 to P change is quite severe, as proline is a non-polar residue, whilst arginine is positively charged. We had predicted that, in addition, the change from arginine to proline would cause conformational rigidity in the protein chain, leading to a significant effect on the local protein structure around the site of mutation, thus leading to functional effects.^25^ Our data shows that this is indeed the case as we do see a more severe effect on the susceptibility to DNA damage in both heterozygous and homozygous R73P variant knock-in cells.

The V58L CFAP410 ALS variant was reported to be more stable than the wildtype protein due to increased phosphorylation by NEK1 and it was suggested that an accumulation of both NEK1 and CFAP410 leads to an aberrant phenotype in motor neurons.^23^ The same variant has recently been shown to lead to increased apoptosis and altered DNA damage response in patient-derived motor neurons.^24^ Our ALS variant knock in neurons showed a trend toward a reduction in the interaction with Nek1 as well as other interactors such as Spata7, suggesting a common mechanism for reduced function variants of CFAP410 in disease. These changes are relatively subtle and may require sensitive techniques to better probe their relative differences. It is interesting to note that although each of the three stressors we applied works through different mechanisms (Na arsenite, reactive oxygen species; Etoposide, double-strand breaks; Valinomycin mitochondrial stress), each resulted in a comparable vulnerability in our Cfap410 variant cells. Because of this, we cannot rule out the increased DNA damage response and susceptibility to genotoxic stress is due to other uncharacterized roles of CFAP410 or ciliary defects – for example, primary cilia can play a role in modulating autophagy, and disruptions to autophagy have also been shown to result in increased sensitivity to etoposide.^42^^,^^43^

In addition, we also found that the DNA damage response (DDR) is affected in the *Cfap410* variant knock in cells. Checkpoint kinase 1 (Chk1), an important mediator and signal transducer in DDR^44^ is activated by phosphorylation. We found that this happens to a lesser extent in the variant knock-in neurons, resulting in an attenuation of the activation of DNA damage checkpoints, possibly leading to the aberrant activation of DNA repair pathways.^45^^,^^46^^,^^47^ It is known that activated Chk1 phosphorylates Cell division cycle 25 (Cdc25) leading to cell-cycle arrest.^48^ The regulation of Cdc25B by Chk1 appears to occur at centrosomes. The phosphorylation of Cdc25B by Chk1 results in its sequestration from the centrosome, leading to the inhibition of centrosomal cyclin-dependent kinase 1 (Cdk1).^49^ Since Cfap410 is a basal body/centrosomal protein, mutations in this protein may affect the localization of Cdcs. Further studies characterizing the effect on DDR pathways and the longer-term effects of CFAP410 variants on genome stability could shed light on the possible mechanisms by which Cfap410 functional variants contribute to ALS risk. DNA damage and repair appears to be a major pathway that is affected in neurodegenerative disorders,^50^^,^^51^ the endogenously tagged variants generated here will provide an excellent platform from which to identify interactors to further dissect the role of CFAP410 in disease.

### Limitations of the study

Our studies show that V20M and R73P mutations in the context of mouse do not affect ciliogenesis or gross localization to the base of the primary cilia but do contribute to an increased sensitivity to DNA damage. Further characterization of the composition of the ciliary structures and their competence to transduce signals is required to determine whether these mutations or other Cfap410 variants affect the many functions of the primary cilia. Although we show disrupted protein-protein interactions with DDR factors, particularly for the R73P variant, we cannot definitely say the loss of these particular interactors results in the DDR phenotype we observe, as there may also be changes to further uncharacterized interactions. Our endogenously tagged Cfap410 variants will allow for further unbiased characterization of this and any changes to the interactome in future studies.

## Resource availability

### Lead contact

Requests for further information and resources should be directed to and will be fulfilled by the lead contact Dr Vasanta Subramanian (bssvss@bath.ac.uk).

### Materials availability

All unique/stable reagents generated in this study are available from the lead contact upon request.

### Data and code availability

• Data: Original western blot images and PCR gel images have been deposited at Mendeley and are publicly available as of the date of publication. The DOI is listed in the key resources table. Microscopy data reported in this article will be shared by the lead contact upon request.
• Code: This article does not report a high-throughput dataset nor original code
• Additional information: Any additional information required to re-analyze the data reported in this article is available from the lead contact upon request.

## Acknowledgments

We would like to acknowledge T. Yamomoto for the Multiplex CRISPR-Cas9 Assembly System Kit and S. Park for the pDisplay plasmid obtained from Addgene. We would like to thank Allan Bradley for the plasmid pPB-CAG.OSKML-pudtk and Shankar Srinivas for the pCagTag plasmid. The following antibodies were obtained from the Developmental Studies Hybridoma Bank, created by the NICHD of the NIH and maintained at The University of Iowa, Department of Biology, Iowa City, IA 52242: 2H3 developed by Jessell, T.M. & Dodd, J. (HHMI/Columbia University), 40.2D6 developed by Jessell, T.M. & Brenner-Morton, S. (HHMI/Columbia University) and Rat401 (Nestin) developed by Hockfield, S. (Yale University School of Medicine). The research funded by a project grant from the 10.13039/501100000406Motor Neurone Disease Association (Project Grant 866-791) to VS.

## Author contributions

The project was conceived by VS. VS and RF designed the experiments, and RF performed the experiments. RF and VS analyzed the data and wrote the manuscript.

## Declaration of interests

The authors declare no competing interests.

## STAR★Methods

### Key resources table

**Table:** 

REAGENT or RESOURCE	SOURCE	IDENTIFIER
**Antibodies**		

Islet1/2	DSHB	Cat# 40.2D6; RRID:AB_528315
Pericentrin	Abcam	Cat# ab4448; RRID:AB_304461
Peripherin	Millipore	Cat# AB1530; RRID:AB_90725
TDP43	ProteinTech	Cat# 10782-2-AP; RRID:AB_615042
Nestin	DSHB	Cat# Rat401; RRID:AB_1118533
ΥH2AX	Biolegend	Cat# 613401; RRID:AB_315794
P53BP1	Novus	Cat# NB100-304; RRID:AB_350221
HA	Covance	Cat# MMS-101P; RRID:AB_2314672
Cleaved caspase3	Abcam	Cat# AB13847;RRID:AB_443014
MitoTracker Red CMXRos	Invitrogen	Cat# M7512
Arl13B	Proteintech	Cat# 17711-1-AP; RRID:AB_2060867
NEK1	Santa cruz	Cat# sc398813; RRID:AB_2885034
SPATA7	Proteintech	Cat# 12020-1-ap; RRID:AB_2195380
FBXO3	Santa cruz	Cat# 514625; RRID:AB_3713047
pCHK1 (Ser317)	Cell Signalling	Cat# 2348; RRID:AB_331212
CHK1	Santa Cruz	Cat# sc8408; RRID:AB_627257
HRP α GAPDH	Abcam	Cat# Ab9482; RRID:AB_307272
AlexaFluor 488 Gt α Ms	Invitrogen	Cat# A-11001; RRID:AB_2534069
AlexaFluor 594 Gt α Rbt	Invitrogen	Cat# A-11008; RRID:AB_143165
AlexaFluor 488 Gt α Rbt	Invitrogen	Cat# A-11012; RRID:AB_141359
AlexaFluor 568 Gt α Rbt	Invitrogen	Cat# A-11011; RRID:AB_143157
AlexaFluor 488 Gt α Rt	Invitrogen	Cat# A-11006; RRID:AB_141373

**Bacterial and virus strains**		

10Beta	NEB	Cat# C3019
Stable	NEB	Cat# C3040

**Chemicals, peptides, and recombinant proteins**		

SB431542	Cayman	Cat# 04-0010-05
CHIR99021	Cayman	Cat#13122
DMH1	Tocris	Cat# 4126
growth factor-reduced Matrigel	Corning	Cat# 354230
FGF2	Peprotech	Cat# 100-18B
EGF	Peprotech	Cat# 315-09
B27 +VitA	Gibco	Cat# 15360284
B27 -VitA	Gibco	Cat# 15440584
DMEM, high glucose, GlutaMAX, pyruvate	Gibco	Cat# 12077549
DMEM/F-12, GlutaMAX™ Supplement	Gibco	Cat# 11514436
E8 media and supplement	Gibco	Cat# 15190617
KnockOut Serum Replacement - Multi-Species	Gibco	Cat# 15621882
Matrigel GFR LDEV free	Corning	Cat# 354230
N2	Gibco	Cat# 17502001
Neurobasal	Gibco	Cat# 11570556
SiMAG-Biotin beads	2BScientific	Cat# 1503-1
TrypLE 1x with phenol red	Gibco	Cat# 10043382
purmorphamine	Cayman	Cat# 10009634
BDNF	Peprotech	Cat# 450-02
GDNF	Peprotech	Cat# 450-10
CNTF	Peprotech	Cat# 450-13
Sodium arsenite	Bdh	—
Etoposide	MP biomedical	Cat# 02193918-CF
Valinomycin	Sigma	Cat# V0627
alpha-MEM with deoxyribonucleosides	Gibco	Cat# 22571020
Trizol	Gibco	Cat# 1559026
iQ5 Sybr green supermix	Biorad	Cat# 170-8880
GoTaq G2 Flexi	Promega	Cat# M7805
T7E1 Endonuclease	NEB	Cat# M0302L
T4 Polynucleotide Kinase	Fisher	Cat# 10120670
T4 DNA Ligase	Fisher	Cat# 10723941
Q5 2x Master Mix	NEB	Cat# M04925
HiFi DNA Assembly mix	NEB	Cat# E2621
OptiMEM	Gibco	Cat# 10149832
Lipofectamine 2000	Invitrogen	Cat# 11668027
Proteinase K	Roche	Cat# 3115879001
Accutase	ThermoFisher	Cat# A1110501
cOmplete mini	Roche	Cat# 11836170001
M-Mulv H-minus reverse transcriptase	ThermoFisher	Cat# EP0451

**Critical commercial assays**		

CellTiter 96® Non-Radioactive Cell Proliferation Assay (MTT)	Promega	Cat# G4000

**Experimental models: Cell lines**		

R1 mESC	Source?	RRID:CVCL_2167
R1 CFAP410^+/HA^	This paper	—
R1 CFAP410^HA/HA^	This paper	—
R1 CFAP410^+/V20M-HA^	This paper	—
R1 CFAP410^V20M-HA/V20M-HA^	This paper	—
R1 CFAP410^+/R73P-HA^	This paper	—
R1 CFAP410^R73P-HA/R73P-HA^	This paper	—

**Oligonucleotides**		

See Table S1 for PCR primers	—	—
See Table S2 for sgRNA sequences and cloning oligos	—	—
See Table S3 for donor assembly primers	—	—

**Recombinant DNA**		

pX330A1x2	Sakuma et al^52^	Addgene #58766
pX330S-2	Sakuma et al.^52^	Addgene #58766
pDisplay-mSA-EFGP-TM	Lim et al.^53^	Addgene #39863
pCagTag	Trichas et al.^54^	Addgene #26771
pBluescript	Stratagene	X52325.1
pPB-CAG.OSKML-pudtk	Yusa et al.^55^	https://doi.org/10.1038/nmeth.1323
pBS-puDtk	This paper	—

**Software and algorithms**		

Fiji	Schindelin. et al.^56^	fiji.sc/
Fiji plugin – BaSiC	Peng et al.^57^	sites.imagej.net/BaSiC/
R version 3.5.3	R core team^58^	www.R-project.org/
R package – ggplot 2 3.2.0	Wickham et al.^59^	ggplot2.tidyverse.org
SPSS 23	IBM	RRID:SCR_002865
LAS AF	Leica	RRID:SCR_013673
Photoshop CS3	Adobe	RRID:SCR_014199

### Experimental model and study participant details

R1 mouse embryonic stem cells (R1 mESCs, male 129 substrain, RRID:CVCL_2167) were cultured on 0.1% gelatin (porcine skin gelatin, Sigma) coated tissue culture dishes (Falcon) in DMEM (Gibco Life Technologies) supplemented with 10% Knockout serum replacement (GibcoLife Technologies), 5% FBS (Labtech), 10 ng/mL Leukemia inhibitory factor (LIF), 1 % non-essential amino acid (NEAA; Gibco), 1μM PD-0325901 (Tocris), 3μM CHIR99021 (Cayman), 0.1 mM β-mercaptoethanol (Sigma). R1 mESCs were sub-cultured every two days using 0.05% trypsin EDTA (Gibco Life Technologies). P19 embryonal carcinoma cells^60^ were cultured in α-MEM (Gibco Life Technologies) supplemented with 10% FBS (Biosera) and 1% NEAA on gelatin coated tissue culture dishes. Media was changed every day and cells were subcultured every two days using 0.05% trypsin EDTA. Neural progenitor cells (NPCs) derived from R1 for expansion were maintained on Matrigel (Corning) coated dishes in DMEM:F12 (Gibco Life Technologies) supplemented with 1x B27 (Gibco Life Technologies), 20ng/ml EGF, 20ng/ml FGF (both from Peprotech). All cell cultures were maintained at 37°C in 5% CO2.

#### Animals

Inbred female and male *C57/Bl6* mice were maintained on a 12 h light/dark cycle with access to food and water *ad libitum*. Pregnant Dams were culled by Schedule1 method and brains isolated for e12.5, e14.5, e16.5, e18.5 as well as from postnatal PN0 and PN15. All animal procedures were reviewed and approved by the Animal Welfare & Ethical Review Body (AWERB) of the University of Bath and performed under approved Project licence (PP1194749) in accordance with UK Home Office guidelines and the UK Animals (Scientific Procedures) Act, 1986.

### Method details

#### Differentiation of P19 cells

Cells were washed with PBS and trypsinised with 0.05% Trypsin EDTA (Gibco Life Technologies). 2.5 × 10^5^ cells/ml were seeded in 90mm suspension dishes (Sterilin) in a-MEM supplemented with 10% KOSR (Gibco Life Technologies), 1% Glutamax and 1% NEAA. Retinoic acid (0.5 μM; Sigma) was added after 24h. After 48h, EBs were plated onto Matrigel (BD)-coated coverslips in a-MEM, 1% Glutamax, 1% NEAA, 1% KOSR and 0.5 μM retinoic acid. This media was changed every other day until day six when it was changed to neurobasal media containing 1x B27 (Gibco Life Technologies), 1% KOSR and 1% Glutamax.

#### Isolation of mouse brain tissue

Pregnant C57BL/6J dams were killed by cervical dislocation prior to dissection for isolating brains from embryonic stages. E18.5 brains were split into fore and mid-brain. Postnatal brains were split into cerebrum and cerebellum. Tissues were snap frozen in Trizol (1ml per 50mg), freeze thawed and disrupted using a Dounce homogeniser.

#### RNA isolation and cDNA synthesis

P19 cells for RT-qPCR were harvested at appropriate time points during differentiation as in^61^ modified from.^62^ Adherent cells were lysed in 1ml/cm^2^ Trizol (Invitrogen), scraped, transferred to a centrifuge tube and briefly vortexed to fully lyse the cells. RNA was isolated from the Trizol lysates following manufacturer’s instructions. RNA yield was determined using Nanodrop spectrophotometer and RNA quality assessed on 1.5% denaturing agarose gel. Equal masses of RNA were re-precipitated and dissolved in equal volumes of DEPC (Sigma) water then treated with RNAse free DNAse (Ambion) at 37°C for 30 min. Quantity and quality were checked again as before. Total RNA was annealed to oligo-dT primers. cDNA synthesis was performed using M-MuLV H-minus reverse transcriptase for 1 h at 42°C and inactivated at 70°C for five minutes (all components from RevertAid™ H Minus Reverse Transcriptase kit, Fermentas). Duplicate reactions substituting the RT enzyme for DEPC treated water were also performed as above for each sample. RT reactions were performed at 42°C for 60min and inactivated by 5min at 70°C. Each reaction was diluted to 10ng/ul of starting material in H2O.

#### Quantitative PCR

A Mastermix comprising iQ SYBR Green supermix (BioRad), water and cDNA was made for quantitative PCR. Appropriate primers were added and divided into three replicates for each gene on PCR plates (Thermo). Final reactions were 20μl in volume with 0.1μM primers and 10ng cDNA (see Table S1 for primer sequences). Reactions were performed in a BioRad iQ5 cycler. Dynamic well factors were collected for 2min 30sec, then forty cycles at 60°C and 95°C for 20s each followed by a melt curve. Expression levels were determined relative to GAPDH from baseline subtracted curves and corrected using primer efficiencies determined previously from serial dilutions of PCR product. qPCR reactions were conducted in triplicate in three independent experiments.

#### Guide design and cloning

Using the mouse *CFAP410* locus (GRCm38 10 7797802-77987405). Guide pairs were designed using chopchop.cbu.uib.no^63^ and crispr.mit.edu.^64^ See main text for rationale. For each edit site, four pairs of guides which showed no joint off-target binding and were proximal to the desired insert site were chosen. See Table S2. Oligos encoding the guide sequences were designed to include overhangs compatible with the multiplex pX330 based nickase Cas9 constructs (gift from T Yamomoto). The oligos comprising each pair of guides were hybridised by cooling from 95^0^ cloned by Golden Gate assembly into the BbsI sites of either pX330A1x2 (sgRNA-A) or pX330S-2 (sgRNA-B) using T4 ligase (Thermo). Were transformed into DH5a. Colonies were screened by colony PCR. Positive clones were confirmed by sequencing from the U6 promoter (Eurofins MWG U6 primer). The sgRNA-B expression cassette from pX330S-2 was transferred into the BsaI sites of the corresponding pX330A1x2 sgRNA-A plasmid again by Golden Gate assembly. Clones were screened by NdeI digest.

#### Cloning of heterozygous alleles for sequencing

Purified Q5 PCR products from potential heterozygous clones to be further characterised were ligated into SmaI digested pBluescript II KS (+) (100ng) in 20μl 1:3 reactions with 1x T4 ligase buffer (Thermo), 5U T4 ligase (Thermo), 10U SmaI (Thermo). Reactions were run on PCR machine with the following cycling parameters: 10 × 20min@25^o^C, 30 × 1min@10^o^C, 30s@22^o^C 30s@4^o^C, 30s@30^o^C, 1h@25^o^C then held at 4^o^C.^65^ Ligations were transformed into DH10B (NEB), and clones analysed by colony PCR using GoTaq G2 Flexi (Promega) in 30μl reactions comprised of 1x ‘Green’ Flexi buffer (Promega), 1.5mM MgCl2, 0.2mM dNTPs (Bioline), 0.5μM of C21_R361Q_HA_F2/R2 and 0.025U GoTaq G2 polymerase. PCRs were performed using the following protocol: 98°C for 2min, then thirty cycles of 98°C for 20s, annealing for 20s and extension at 72°C (see Table S1 for details), followed by a final extension at 72°C for 5min. Completed reactions were digested in 50ul with 1x EcoRI buffer and 0.5U EcoRI (Thermo) for 1h at 37^0^. Variant clones from each transformation were analysed by sequencing using the T7 promoter.

#### Cloning of genomic donor targeting vectors

To create the donor plasmid backbone pBS-puDtk, the PGK Puro-deltaTK cassette of pPB-CAG.OSKML-puDtk (gift from A Bradley)^55^ was amplified by PCR using Q5 HiFi polymerase (NEB) and primers containing SapI sites with variable overhang homologous to that of the SapI site of pBluescript II KS (+) (See Table S3 for sequences). PCR products and pBluescript were digested with SapI, gel purified and ligated using T4 ligase (Thermo) and transformed into DH10b.

Homology arms derived by PCR from the BAC clone bMQ207-G4 from the 129S7/AB2.2 BAC library (Source Bioscience). The intended mutation or epitope tag, a novel synonymous restriction site and a mutated PAM site were included in overlapping central primers while outer primers were appended with the sequences flanking the EcoRV site of pBS-puDtk. See primers listed in Table S3. The PCR cycling parameters used were 1x Q5 (NEB), 0.5μM of forward and reverse primer and 5ng of BAC in a 50μl reaction. PCR products and EcoRV linearised pBS-puDtk were purified by gel excision and assembled in a 20μl reaction with 50ng of vector and 100ng of each homology arm fragment and 1x HiFi DNA Assembly mix (NEB). Reactions were transformed in NEB Stable cells.

Correct assembly was confirmed by sequencing using the T7 and M13 promoter primers (stock primers, Eurofin MWG) and across the central join using primers upstream of the HA, V20M and R73P donors respectively.

#### Screening of guides

R1 mESCs cultures at 70-80% confluency were fed with fresh media two hours prior to transfection. Single cell suspensions of R1 mESCs were transfected following the method of Tamm et al.,^66^ pX330A1x2 nCas9 containing the guide pairs to be screened was used at 0.5ug per replicate on a 24well plate. DNA was complexed with Lipofectamine 2000 (Invitrogen) in OptiMEM (Gibco) at a ratio of 1:4 (Reagent:DNA) for 50μl per replicate. Cells were trypsinised to single cells, resuspended in complete medium and counted by haemocytometer. The appropriate numbers of cells in suspension was centrifuged at 270g for five minutes, resuspended in OptiMEM and centrifuged again. The final pellet was resuspended in OptiMEM to a final concentration of 10^5^ cells per 50μl OptiMEM and mixed with 50μl DNA Lipofectamine solution and incubated at room temperature for five minutes. Cells were co-transfected with pCagTag (Gift from S Srinivas)^54^ to determine transfection efficiency. The transfected cell suspension was transferred to pre-warmed media on gelatinised 24well plates with 500μl complete medium per well. Transfected cells were analysed after 24h. Transfection efficiency was determined by fixing two wells for each primer pair with 4% PFA for ten minutes at room temperature, cells were counterstained with DAPI and the proportion of GFP expressing cells quantified from four different fields per condition. For determining sgRNA efficacy, duplicate wells were lysed for DNA extraction by proteinase K based methods.

#### Isolation of genomic DNA isolation from mammalian cells

DNA was prepared using either the HotShot method^67^ or by lysis in Proteinase K (Roche) containing buffer followed by phenol:chloroform extraction as indicated For HotShot method, media was aspirated and cells were washed with PBS before adding 150μl/cm2 of lysis buffer (25 mM NaOH, 0.2 mM EDTA, both Sigma). Samples were incubated at 95°C for 15min then cooled briefly on ice before adding equal volume of neutralising buffer (40mM Tris-HCl, Sigma).

For proteinase K based DNA isolation, media was aspirated and cells were washed with PBS before adding 150μl/cm2 of lysis buffer (0.2 mg/ml proteinase K (Roche), 100mM NaCl, 0.5M EDTA, 0.5% SDS in 10mM Tris pH 8.0, all Sigma). After incubation overnight at 50°C lysates were transferred to microfuge tubes with equal volumes of phenol:chloroform:isoamyl alcohol (PCI, 25:24:1, MP biomedical, Sigma, Sigma). After mixing and centrifuging for five minutes at 16,000g, the aqueous phase was recovered and precipitated with 0.2M NaCl (final) and 75% ethanol (final). Precipitated DNA was pelleted at 16,000g for 15min, the supernatant discarded and replaced with 70% ethanol. After a further 10min centrifugation, the supernatant was discarded again and the pellet dried. DNA was dissolved in 200μl/cm2 TE (1mM EDTA in 10mM Tris pH 8.0, both Sigma).

#### T7E1 assay

The fraction of modifications introduced by CRISPR/Cas9 to the targeted site was determined by T7 endonuclease 1 digestion of the resulting heteroduplexes formed after PCR and subsequent rehybridization.^68^ Primers to amplify asymmetrically across the intended edit point were designed and optimised by gradient PCR using Q5 high fidelity Taq mix (NEB). PCRs were performed using 10ng of DNA prepared by proteinase K and PCI extraction in 50μl with 0.5μM primers. PCR conditions were 95°C for 2min followed by thirty cycles of 95°C 10s, Ta 10s, 72°C 20s, 72°C 5min then 10°C hold See Table S1 for annealing temperatures and primer sequences. PCR products were purified by spin column (QIAquick PCR Purification Kit, Qiagen) and eluted in sterile water. 200ng of the PCR product was diluted in NEB buffer 2 and rehybridised by heating to 95°C for five minutes then lowering the temperature from 95-85°C at -2°C/s then 85-25°C at -0.1°C/s. 10U of T7E1 (NEB) or water (as control) was added to each reaction to bring the final volume to 20μl and the reactions incubated at 37 for 15 min. EDTA was added to a final concentration of 25mM to stop the reaction. Reactions were electrophoresed on 2% TBE agarose gels and band intensity quantified using ImageJ. Gene edited fractions were calculated as % gene modification = 100 × (1 − (1 − fraction cleaved)1/2) from three independent experiments.

#### Electroporation of ES cells

R1 mESCs were washed with PBS and trypsinised to single cells, resuspended in complete medium and counted using a haemocytometer.

For electroporations using the Bio-Rad genepulser, 5 × 10^6^ cell were centrifuged at 270g for 5min, resuspended in PBS and centrifuged again. Cells were resuspended in 800μl of PBS containing 25μg of the pBS PuroDtk donor plasmid, 2.5μg pX330A containing the corresponding opitimal guide pair and 2.5μg pDisplay mSA EGFP TM (gift from Sheldon Park, Addgene plasmid 39863). The suspension was then transferred to 1ml 2mm path cuvettes (Biorad) and electroporated at 300V, 250μF.

For electroporations using the NEPA21 electroporator (Nepagene), 1 × 10^6^ cell were centrifuged at 270g for 5min, resuspended in PBS and centrifuged again then resuspended in OptiMEM (Gibco) and centrifuged again. Cells were finally resuspended in 100μl of OptiMEM containing 10μg of the pBS PuroDtk donor plasmid, 2.5μg pX330A containing the corresponding guide pair and 2.5μg pDisplay mSA EGFP TM (Lim et al.,^53^). The suspension was then transferred to 1ml 2mm path cuvettes (Nepagene) and electroporated using two poring pulses (+) at 135V for 5ms with 50ms intervals and a 10% decay rate followed by five transfer pulses (±) at 20V for 50ms with 50ms intervals with a 40% decay rate.

Electroporated cells were diluted 1:100 in complete ES medium medium and seeded at 1ml/10cm^2^. 18h after electroporation, media was changed to include 0.5μg/ml puromycin (Sigma). Selection was maintained for 48h after which magnetic sorting was performed. Anti-HA beads (Pierce) were washed in and kept in PBS with 0.1% BSA (PBS-BSA). Cells were trypsinised and centrifuged at 270g for 5min, resuspended in PBS-BSA and spun down again. The cell pellet was resuspended in the 1ml of HA-bead suspension and incubated rocking for five minutes. The beads were pelleted by magnet for one minute after which the supernatant was aspirated and the pellet resuspended in PBS-BSA. This was repeated once more and the pellet resuspended in complete medium. Sorted cells were seeded at a density of 750 cells per 10cm^2^ on gelatinised dishes in complete medium. Only 50% media changes were made after 24-36h until colonies had established. Once large enough, colonies were picked to individual wells of a 24 well plate and expanded for characterisation.

#### High resolution melt curve analysis

PCRs were performed on 50ng of genomic DNA isolated form individual ES clones using GoTaq G2 Flexi (Promega) in 50μl reactions containing 1x colourless GoTaq reaction buffer, 0.2mM dNTPs (Bioline), 0.75U GoTaq polymerase, 0.5x EvaGreen (Biotium), assay dependent concentrations of MgCl_2_ (1.0μM and 1.2μM for V20M and R73P, respectively) and primers (See Table S1 for primer sequences). PCR cycling conditions were 3min at 95°C followed by 30 cycles of 95°C for 15s, T_a_ for 15s and 72°C for 20s then a final extension at 72°C for 5min (See Table 1 for T_a_). Where digestion of the PCR product was performed, reactions were divided in two aliquots and 0.5U of Tru1I (MseI) (Thermo) added in 10μl reaction volume containing 1x Buffer R. Digests were incubated at 65°C for 1h. Melt curves were performed on a Biorad iQ5 with 0.1°C increments and 15s dwell time.

#### Differentiation of mouse ES cells

mESCs were trypsinised at 60-70% confluence, resuspended in the appropriate complete medium (ESC or NPC) and counted by haemocytometer. Cells well diluted to 10^5^ cells/ml in DMEM:F12 with 10% KOSR, 1μM DMH1 (Tocris), 1μM SB (Tocris), 1% GlutaMax and 0.1 mM β-mercaptoethanol (Sigma), and plated in suspension in non-TC treated 10cm dishes (Sterilin) to form aggregates. Media was changed on day two. On day four, aggregates were collected in centrifuge tubes washed with PBS and disassociated using Accutase (Gibco; 3ml/10cm) for 30min at 37°C. NPC medium [ DMEM:F12 (Gibco) supplemented with 1x B27 (Gibco), 20ng/ml EGF and 20ng/ml FGF] was added and cells were centrifuged at 270g for 3 minutes. After aspirating the supernatant, the pellets were resuspended in NPC medium, gently triturated, and counted by haemocytometer. They were then replated onto Matrigel coated dishes at a density of 10^4^ cells/cm^2^ in NPC medium. After 72h of culture the NPCs were passaged using Accutase as before but with only a 3 minute incubation. NPCs were either expanded for characterisation or progressed to motor neuron differentiation. For motor neuron differentiation, media was changed 24h after passaging of NPC in DMEM:F12 supplemented with 1x B27, 1x GlutaMax, 1μM Retinoic acid (Sigma), 1μM Purmorphamine (Tocris) and 20ng/ml BDNF, GDNF and CNTF (all Peprotech). After a further 48h culture, neurons were washed with PBS and incubated for 30min with Accutase. Neurons were resuspended in MN medium, centrifuged at 270g for 3 minutes. After aspirating the supernatant, the pellets were resuspended in MN medium, gently triturated, and counted by haemocytometer. They were then replated onto poly-l-ornithine and laminin (both Sigma) coated dishes or coverslips at a density of 10^5^ cells/cm^2^ in MN medium.

#### Colony forming assays

Cells were trypsinised at 60-70% confluency, resuspended in the appropriate complete medium (ESC or NPC) and counted by haemocytometer. For ESCs, 2ml of medium was pre-warmed on gelatin coated 12well plates (Falcon). Cells were diluted to 1500 cells/ml and 100μl dispensed to the 12 well plates for a final concentration of 150 cells per well in triplicate. Media was changed after 48h, then every 24h until the fifth day. For NPCs, non-TC treated 12well plates (Falcon) were coated with 0.5% agar (in 1x DMEM:F12). 2x stocks of agar and DMEM:F12 were made and autoclaved or sterile filtered respectively before combining for use. 2x NPC medium was combined with 0.6% agar for final concentrations of 0.3% agar, 20ng/ml EGF (Peprotech), 20ng/ml FGF (Peprotech), 1x B27 (Gibco), 1x GlutaMax (Gibco), 1x DMEM:F12 (Gibco) and kept at 40°C in a water bath. Cells were diluted to 1500 cells/ml and 100μl dispensed to the 12 well plates for a final concentration of 150 cells per well in triplicate. 1ml of agar media was added to each well gently mixed by pipette and allowed to cool at room temperature for 20min. 1ml of complete medium was added on top before returning to the cell culture incubator. Media was changed every 48h for eight days.

The colonies formed after 8 days of culture were washed with PBS, fixed in 4% PFA for 30min RT and stained with 0.01% crystal violet (BDH) for 30min. Stained cells were washed with water until the background was clear. Plates were imaged on a UVP Gel-doc IT2 Imager and colony frequency and area were measured using Fiji. Data was collected from three replicates from three independent experiments.

#### Growth assays

Cells were trypsinised at 60-70% confluence, resuspended in the appropriate complete medium (ESC or NPC) and counted by haemocytometer. Cells were diluted to 3.2 × 10^4^ cells/ml in complete medium. 100μl per well of cell suspension was plated in triplicate for each cell line for each assay interval on 96 well plates for 1 × 10^4^cells/cm^2^. Growth was assayed using the Non-Radioactive Cell Proliferation Assay (Promega) at 24h intervals. Cells were incubated with 15μl/well of dye solution for 2h followed by 100μl of stop solution. After solubilisation for 1h, absorbance was recorded at 570nm with a 700nm reference wavelength.

#### Kill curve assays

Cells were treated with varying concentrations of Etoposide (MP Biomedical), Valinomycin (Sigma) and Sodium Arsenite (BDH). Cells were trypsinised at 60-70% confluence, resuspended in the appropriate complete medium (ESC or NPC) and counted by haemocytometer. Cells were diluted to 19.2 x 10^4^ cells/ml in complete medium. Cell suspension (50μl per well) was plated in triplicate for each cell line on 96 well plates for 3 × 10^4^cells/cm^2^. A dilution series of the appropriate drug in solvent was created such that each was added to complete medium for a final solvent concentration of 0.1% (DMSO) across the series. Drugs were diluted to 2x the indicated concentration in complete medium and added as 50μl per well for the final 1x concentration. Concentrations used were a 1:3 series from 100-0.14μM for etoposide and valinomycin, and 30.0-0.04 μM for sodium arsenite. Viability was determined after 24h using the Non-Radioactive Cell Proliferation Assay (Promega), as described in ‘growth assays’.

#### Drug treatment of EC, NPCs and motor neurons

ESC and NPC were plated at 3 × 10^4^cells/cm^2^ as described above. Neuronal cultures were differentiated as described and replated at a density of 10^5^ cells/cm^2^ in MN medium onto poly-l-ornithine and laminin (both Sigma) coated coverslips for immunostaining, or coated T75s for protein lysates. Etoposide, valinomycin and sodium arsenite concentrations used represent the [EC50] for the heterozygous V20M genotype for each cell type. Neurons: 2 μM etoposide, 0.5μM sodium arsenite, 2μM Valinomycin. After 16h treatment, cells were fixed for immunostaining, or lysed for Co-IP/Western blot.

#### Western blot

Cells were lysed in RIPA buffer (50mM Tris pH7.4, 150mM NaCl, 1% NP40, 0.5% sodium deoxycholate, 0.1% SDS, 1mM EDTA, 1mM PMSF) with 1x cOmplete Mini Protease Inhibitors (Roche). Protein concentration was determined using the BCA assay (Pierce).

For blotting, 50μg of lysate were run on a 12% tris-tricine gel.^69^^,^^70^ Proteins were transferred to 0.22μm PVDF (Millipore) at 50V for 4h in transfer buffer 25mM Tris, 192mM glycine, pH 8.3, 20% methanol. Membranes were blocked in 5% milk (Marvel) in PBS with 0.1% Tween (PBSTw) for one hour and incubated overnight at 4°C with primary antibodies diluted in block. Blots were washed four times for ten minutes each in PBSTw before incubation with Horseradish peroxidase (HRP) conjugated secondary antibodies for one hour at RT. See Table S4 for antibody dilutions. After a further four ten minute PBSTw washes and two PBS washes, blots were incubated with lab made ECL reagent^71^ and imaged on a Fusion SL (Vilber Lourmat).

#### Immunoprecipitation

Cells were lysed in 100μl/cm2 of buffer comprised of 20mM Tris (pH7.5) with 150mM NaCl, 1mM EDTA, 1mM EGTA, 1% Triton X-100, 1mM PMSF (all Sigma) and 1x cOmplete Mini EDTA-free Protease Inhibitor Cocktail (Roche). All steps were done on ice or 4°C with precooled material. Cells were scraped to collect and rocked for 30min before centrifugation at 10,000g for 20min. The supernatant was recovered and protein concentration estimated by BCA assay (Pierce). 2mg of lysate was incubated overnight on a rotator with 50μg Anti-HA magnetic beads (Pierce). Beads were washed three times with PBSTw and eluted twice with 20μl Laemmli buffer and pooled.

#### Immunostaining

Cells cultured on coverslips were washed with PBS and fixed for 15min in 4% PFA. PFA was washed off with two PBS washes and cells dehydrated to 70% ethanol through 30% and 50% for ten minutes each. After rehydration to PBS, wells were incubated in block for 1h RT (PBS with 1% FBS, 0.1% gelatin and 0.5% Triton X-100). Incubation with primary antibody in PBST (PBS with 0.1% Tween) with 0.1% FBS was performed overnight at 4°C. After four PBST washes for ten minutes each, cells were incubated with secondary antibodies together 4′,6-diamidino-2-phenylindole (DAPI, Sigma). See Table S4 for antibody dilutions. Z-stacked images were acquired with either a Leica DM5500B microscope, DFC 360FX camera and LAS software and deconvoluted or using a Zeiss LSM510META confocal microscope using LSM 5 Series software.

#### Quantification of nuclear phospho-ΥH2AX levels

Images were acquired as 3D stacks from four random positions for each condition from three independent experiments. Stacks were deconvoluted in LAS AF. Deconvoluted stacks were subjected to flat-field correction using BaSiC in Fiji,^56^^,^^57^ followed by maximum intensity projection. Nuclear masks were created using the DAPI channel. Median grey values for each nuclei were measured for the channel to be quantified.

### Quantification and statistical analysis

Each experiment was repeated at least three times using at least two independent cell lines of the same genotype. Images were acquired from at least five random fields in each experiment. Images for quantification were acquired at equal gain and exposure, flat field corrected using the BaSiC ImageJ plugin^57^ and further analysed using Fiji as described in the detailed methods. Where possible datapoints representing the quantification of result from each independent experiment are presented dots plotted over bar graphs showing overall mean ± SEM. Where fluorescent intensity analyses have been performed, plots based on median single cell fluorescent intensity are presented. See figure legends for more specific details. Normality was determined using the D'Agostino-Pearson test. Normal data was tested directly using ANOVA and Bonferroni post-hoc. Statistical analysis was performed using either SPSS 23 or R.
