# Supplementary material for: Functional variants of CFAP410 affect the DNA damage response leading to motor neuron degeneration – Implications for ALS
Source: iScience. 2025 Aug 9;28(9):113338. doi: 10.1016/j.isci.2025.113338 (PMC12419110; doi:10.1016/j.isci.2025.113338)

iScience, Volume *28*

Supplemental information

Functional variants of CFAP410 affect the DNA damage response leading to motor neuron degeneration – Implications for ALS

Ross Ferguson and Vasanta Subramanian

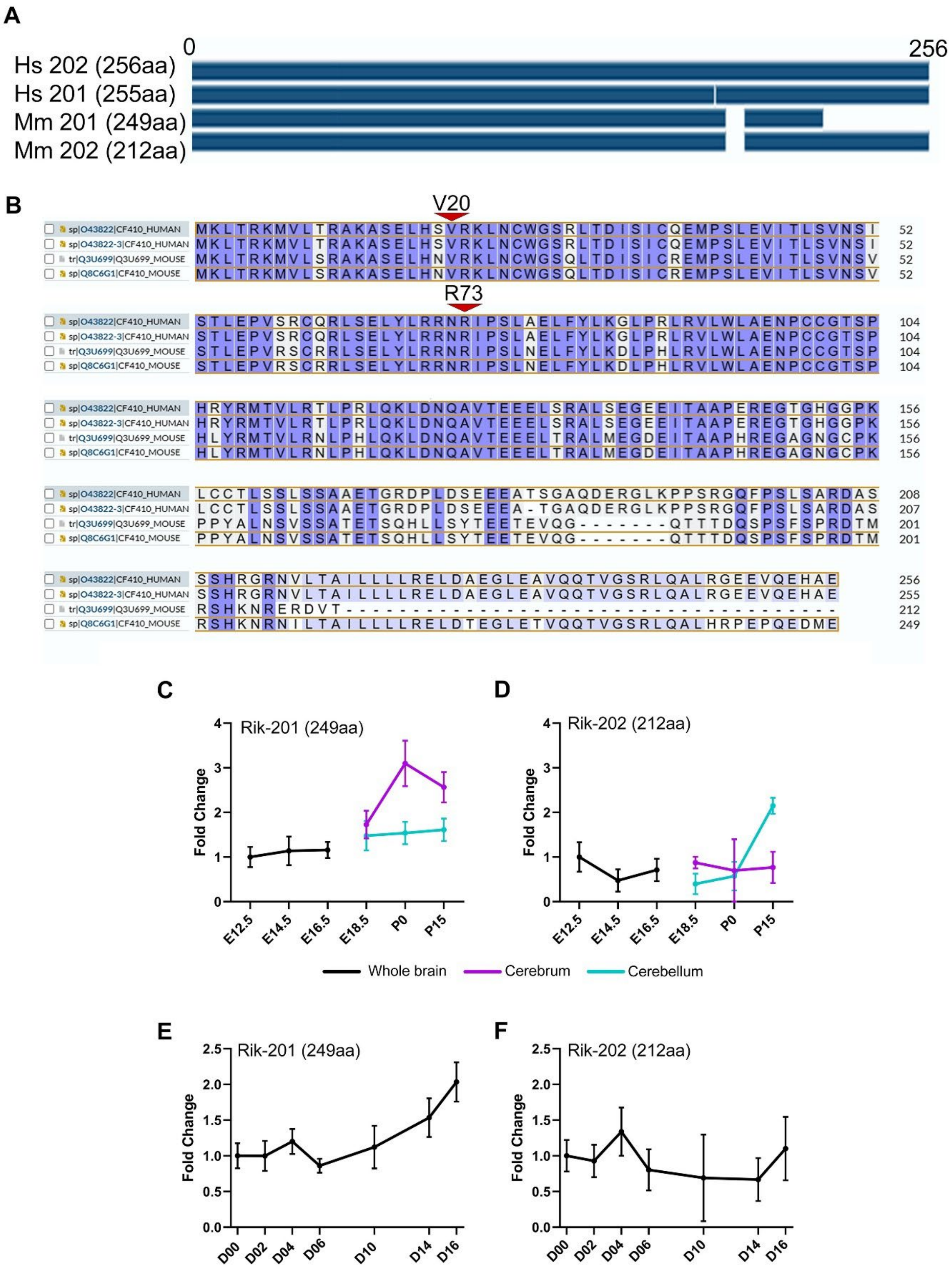
A
0
256
Hs 202 (256aa)
Hs 201 (255aa)
Mm 201 (249aa)
Mm 202 (212aa)
B
V20
R73
sp|O43822|CF410_HUMAN MKLTRKMVLTRAKASELHSVRKLNCWGSRLTDISICQEMPSLEVITLSVNSI 52
sp|O43822-3|CF410_HUMAN MKLTRKMVLTRAKASELHSVRKLNCWGSRLTDISICQEMPSLEVITLSVNSI 52
tr|Q3U699|Q3U699_MOUSE MKLTRKMVLSRAKASELHNVRKLNCWGSQLTDISICREMPSLEVITLSVNSV 52
sp|Q8C6G1|CF410_MOUSE MKLTRKMVLSRAKASELHNVRKLNCWGSQLTDISICREMPSLEVITLSVNSV 52
sp|O43822|CF410_HUMAN STLEPVSRCQRLSELYLRRNRIPSLAELFYLKGLPRLRVLWLAENPCCGTSP 104
sp|O43822-3|CF410_HUMAN STLEPVSRCQRLSELYLRRNRIPSLAELFYLKGLPRLRVLWLAENPCCGTSP 104
tr|Q3U699|Q3U699_MOUSE STLEPVRSCRRLSELYLRRNRIPSLNELFYLKDLPHLRVLWLAENPCCGTSP 104
sp|Q8C6G1|CF410_MOUSE STLEPVRSCRRLSELYLRRNRIPSLNELFYLKDLPHLRVLWLAENPCCGTSP 104
sp|O43822|CF410_HUMAN HRYRMTVLRTLPRLQKLDNQAVTEEELSRALSEGEEITAAPEREGTGHGGPK 156
sp|O43822-3|CF410_HUMAN HRYRMTVLRTLPRLQKLDNQAVTEEELSRALSEGEEITAAPEREGTGHGGPK 156
tr|Q3U699|Q3U699_MOUSE HLYRMTVLRNLPHLQKLDNQAVTEEELTRALMEGDEITAAPHREGAGNGCPK 156
sp|Q8C6G1|CF410_MOUSE HLYRMTVLRNLPHLQKLDNQAVTEEELTRALMEGDEITAAPHREGAGNGCPK 156
sp|O43822|CF410_HUMAN LCCTLSSLSSAAETGRDPLDSEEEATSGAQDERGLKPPSRGQFPSLSARDAS 208
sp|O43822-3|CF410_HUMAN LCCTLSSLSSAAETGRDPLDSEEEA-TGAQDERGLKPPSRGQFPSLSARDAS 207
tr|Q3U699|Q3U699_MOUSE PPYALNSVSSATETSQHLLSYTEETEVQG-------QTTTDQSPSFSPRDTM 201
sp|Q8C6G1|CF410_MOUSE PPYALNSVSSATETSQHLLSYTEETEVQG-------QTTTDQSPSFSPRDTM 201
sp|O43822|CF410_HUMAN SSHRGRNVLTAILLLLRELDAEGLEAVQQTVGSRLQALRGEEVQEHAE 256
sp|O43822-3|CF410_HUMAN SSHRGRNVLTAILLLLRELDAEGLEAVQQTVGSRLQALRGEEVQEHAE 255
tr|Q3U699|Q3U699_MOUSE RSHKNRERDVT------------------------------------- 212
sp|Q8C6G1|CF410_MOUSE RSHKNRNILTAILLLLRELDTEGLETVQQTVGSRLQALHRPEPQEDME 249
C
Rik-201 (249aa)
Fold Change
0
1
2
3
4
E12.5
E14.5
E16.5
E18.5
P0
P15
D
Rik-202 (212aa)
Fold Change
0
1
2
3
4
E12.5
E14.5
E16.5
E18.5
P0
P15
Whole brain
Cerebrum
Cerebellum
E
Rik-201 (249aa)
Fold Change
0.0
0.5
1.0
1.5
2.0
2.5
D00
D02
D04
D06
D10
D14
D16
F
Rik-202 (212aa)
Fold Change
0.0
0.5
1.0
1.5
2.0
2.5
D00
D02
D04
D06
D10
D14
D16

Figure S1 – CFAP410 isoforms conserved between species and are expressed in the developing brain and differentiating neurons.

Related to Figure 1. Alignment overview of mouse and human CFAP410 homologs (A) and residue conservation (B). Ensembl (transcript) and protein (Uniprot) identifiers – Hs 202, human transcript CFAP410-202 encoding O43822; Hs 201, human transcript CFAP410-201 encoding O43822-3; Mm 201, mouse transcript Rik 201 encoding Q3U699; Mm 202, mouse transcript Rik 202 encoding Q8C6G1. qRT-PCR for transcript 1 encoding the 249aa isoform (C) or transcript 2 encoding the 212aa isoform (D) of CFAP410. Data from the whole brain of developing embryos at the indicated stage between E12.5 and E16.5. Between E18.5 to P15, brains were divided into cerebrum and cerebellum. qRT-PCR for transcript 1 (E) or 2 (F) over a time course of differentiating pluripotent cells with samples prepared on the indicated days post-initiation. All data points represent the mean of three replicate experiments each with three technical qPCR replicates. Data normalised to *Ywhaz* expression. Error bars represent ±SEM.

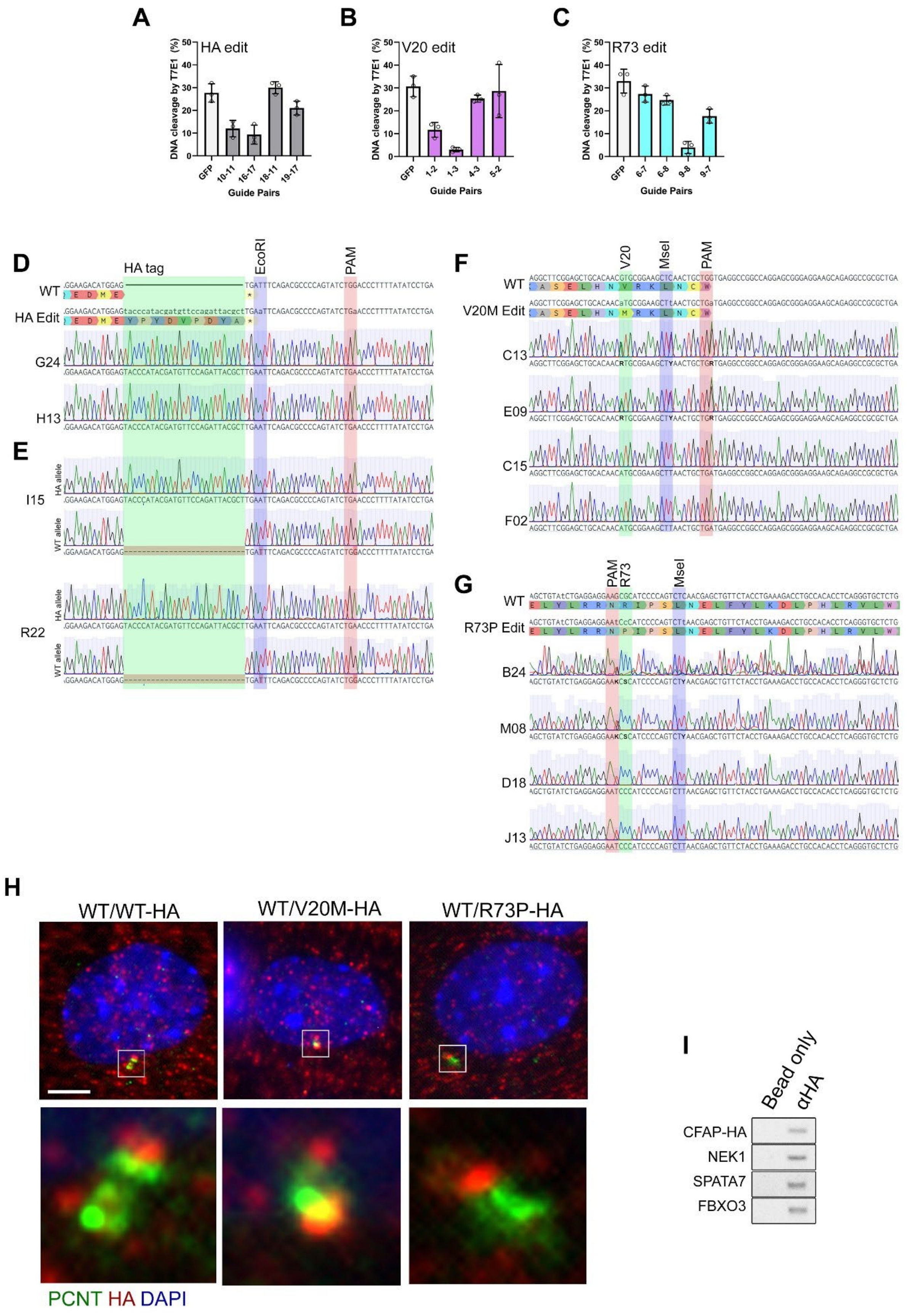
A
B
C
HA edit
V20 edit
R73 edit
DNA cleavage by T7E1 (%)
Guide Pairs
GFP
10-11
16-17
18-11
19-17
1-2
1-3
4-3
5-2
6-7
6-8
9-8
9-7
D
HA tag
EcoRI
PAM
WT
HA Edit
G24
H13
E
HA allele
WT allele
I15
R22
F
V20
MseI
WT
V20M Edit
C13
E09
C15
F02
G
R73
R73P Edit
B24
M08
D18
J13
H
WT/WT-HA
WT/V20M-HA
WT/R73P-HA
PCNT HA DAPI
I
Bead only
αHA
CFAP-HA
NEK1
SPATA7
FBXO3

Figure S2 – CRISPR/Cas9 guide efficiency assays and edited clone sequences.

Related to Figure 1. T7E1 assays to determine guide efficiency in R1 mESC stably expressing GFP (as part of CAG TAG). Single guide targeting GFP and wtCas9 used as inter assay control. Guides assayed as pairs targeting the end of isoform 1 CDS (A), V20 (B) or R73 (C). Bars represent mean of three experiments ±SEM, data points represent experimental means. Sequencing across the Stop / HA tag locus in clones homozygous for the HA tag (D). Sequencing across the stop / HA locus in alleles subcloned into bluescript from clones heterozygous for the HA tag (E). (F) Sequencing across the V20M edit locus in clones identified as either homo- (C15 & F02) or heterozygous (C13 & E09) for the mutation. (G) Sequencing across the R73P edit locus in clones identified as either homo- (D18 & J13) or heterozygous (B24 & M08) for the mutation. (H) Immunostaining for PCNT and HA in representative clones counterstained with DAPI. Inset shows close crop of centriole & basal body. Scale bar 5μm. (I) No bead controls for CFAP410 interactor IPs run alongside R1 CFAP410 WT-HA/WT-HA lysates.

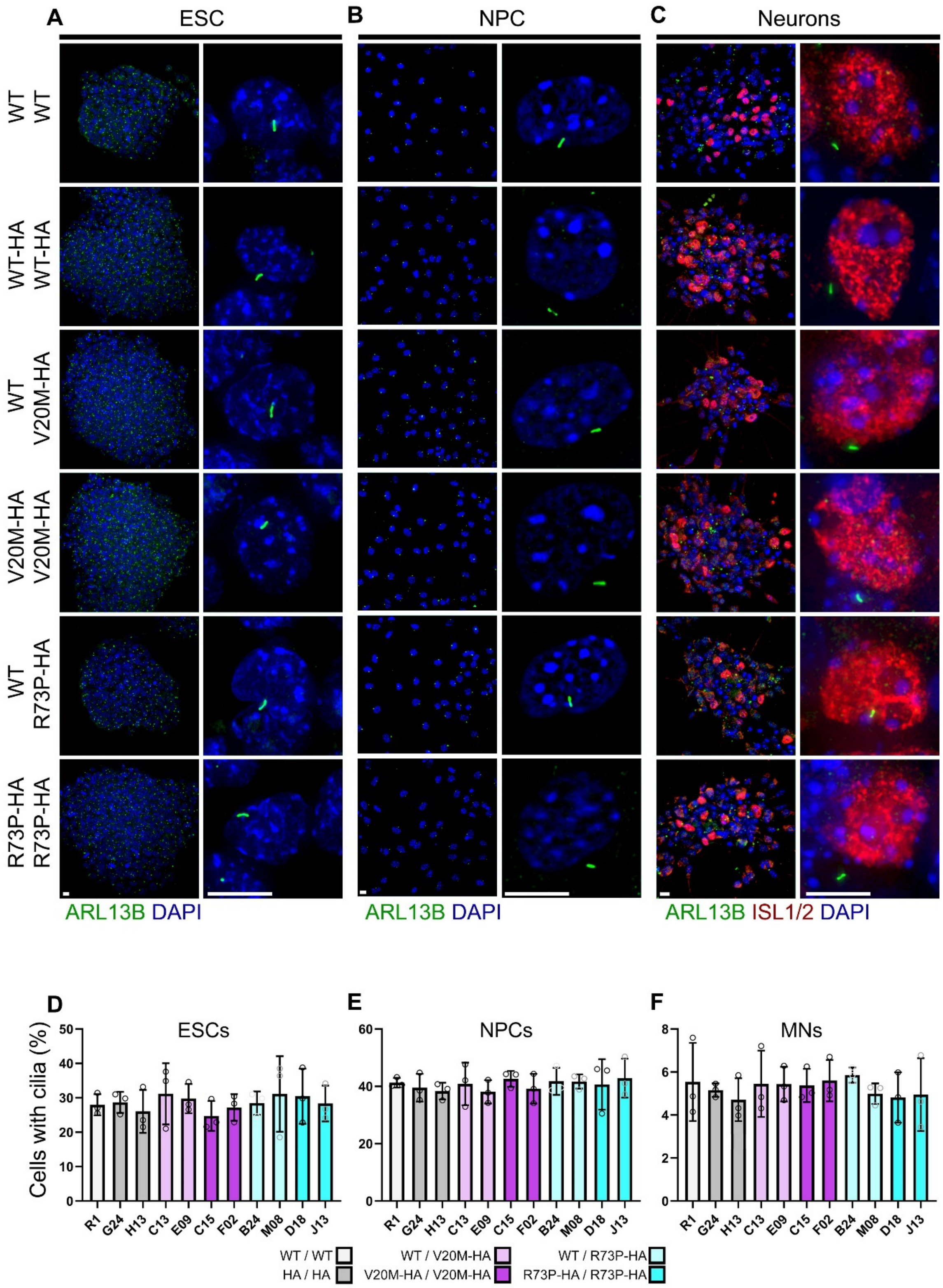

A
ESC
B
NPC
C
Neurons
WT WT
WT-HA WT-HA
WT V20M-HA
V20M-HA V20M-HA
WT R73P-HA
R73P-HA R73P-HA
ARL13B DAPI
ARL13B DAPI
ARL13B ISL1/2 DAPI
D
ESCs
Cells with cilia (%)
E
NPCs
F
MNs
R1
G24
H13
C13
E09
C15
F02
B24
M08
D18
J13
WT / WT
HA / HA
WT / V20M-HA
V20M-HA / V20M-HA
WT / R73P-HA
R73P-HA / R73P-HA

Figure S3 - CFAP410 variants do not affect primary cilia frequency.

Related to Figure 2. Immunostaining for ARL13B in ESCs (A) and NPCs (B), or ISL1/2 and ARL13B in differentiated neurons, scale bars 10µm (C). The frequency of cells with primary cilia was quantified from four random fields each from three experiments (Bar represent mean ±SEM, data points represent experimental medians) for ESC (D), NPC (E) and motor neurons (F). No significant differences found. Data compared by ANOVA with Bonferroni's *post hoc* test.

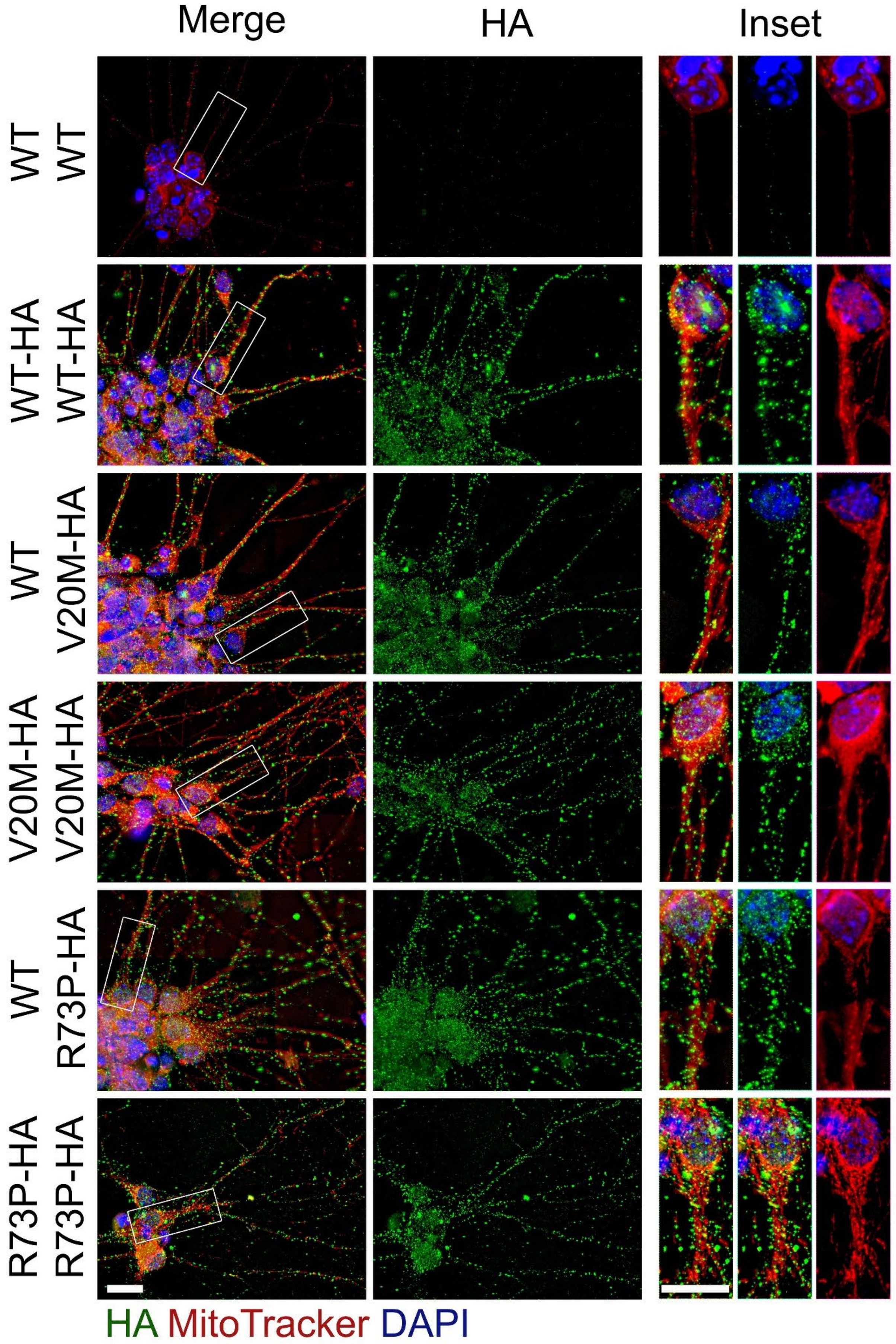
Merge
HA
Inset
WT WT
WT-HA WT-HA
WT V20M-HA
V20M-HA V20M-HA
WT R73P-HA
R73P-HA R73P-HA
HA MitoTracker DAPI

Figure S4 – Immunostaining for HA tag in Mitotracker treated neurons

Related to Figure 2. Immunostaining for HA tagged CFAP410 in neurons differentiated from CFAP410 variant ESCs. Representative images from a single clone of each genotype shown. Scale bar 10µm. Inset shows a single neuronal nuclei and soma.

Key to genotypes: WT/WT: $CFAP410^{WT/WT}$ (R1); HA/HA: $CFAP410^{WT\text{-}HA/WT\text{-}HA}$ (G24); WT/V20M-HA: $CFAP410^{WT/V20M\text{-}HA}$ (C13); WT/R73P-HA: $CFAP410^{WT/R73P\text{-}HA}$ (B24); V20M-HA/V20M-HA: $CFAP410^{V20M\text{-}HA/V20M\text{-}HA}$ (C15); R73P-HA/R73P-HA: $CFAP410^{R73P\text{-}HA/R73P\text{-}HA}$ (J13).

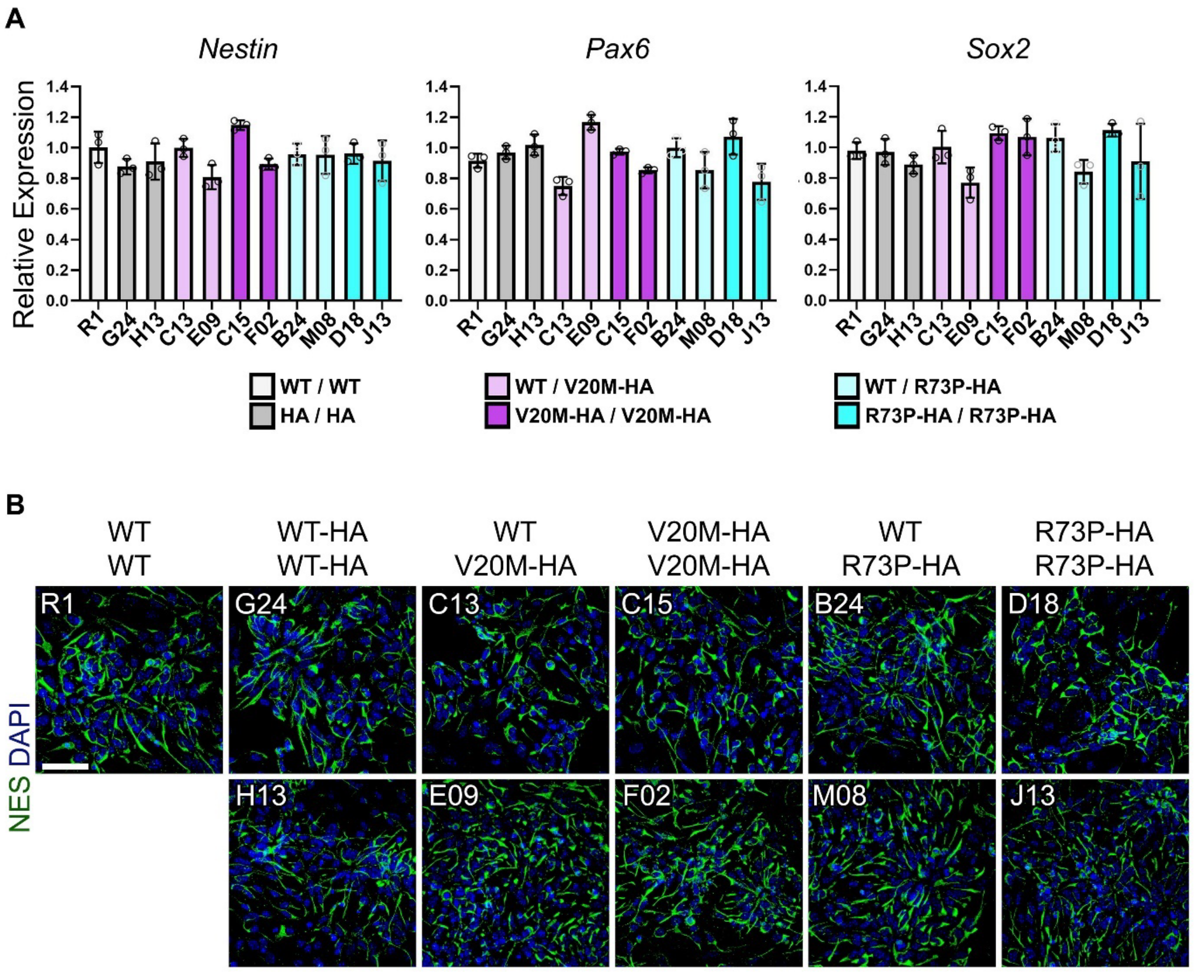

Figure S5 – CFAP410 variant NPCs show no significant changes in expression of key markers

Related to Figure 3. qRT-PCR analysis of *Nestin*, *Pax6* and *Sox2* expression in passage 2 NPCs differentiated from R1 and CFAP410 variant ESCs (A). All data points mean of three replicate experiments each with three technical qPCR replicates. Data normalised to *Ywhaz* expression. Mean shown with error bars ±SEM. Immunostaining for nestin expression in the same NPCs (B). Scale bar 50µm.

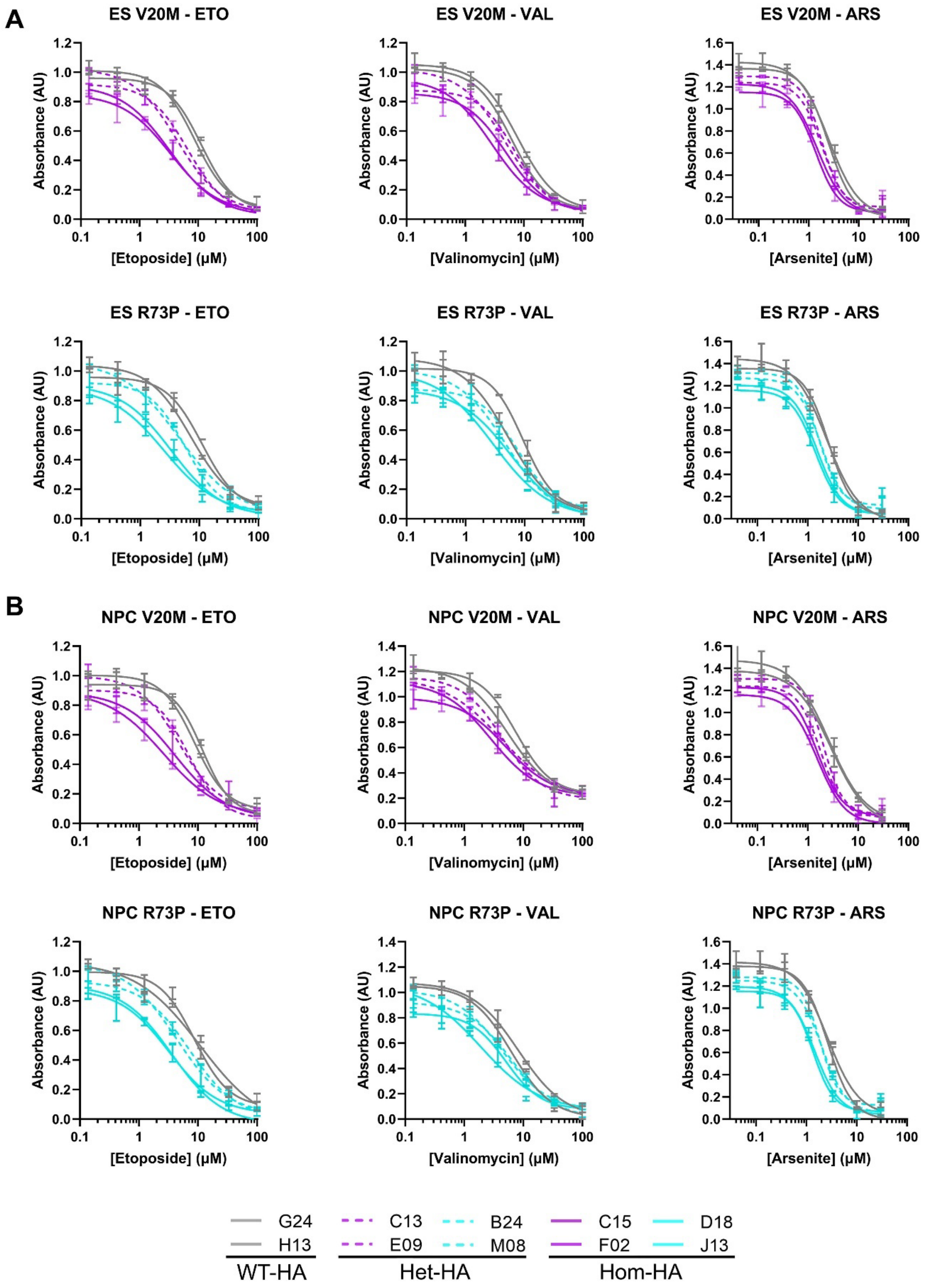
A
ES V20M - ETO
ES V20M - VAL
ES V20M - ARS
ES R73P - ETO
ES R73P - VAL
ES R73P - ARS
B
NPC V20M - ETO
NPC V20M - VAL
NPC V20M - ARS
NPC R73P - ETO
NPC R73P - VAL
NPC R73P - ARS
Absorbance (AU)
[Etoposide] (μM)
[Valinomycin] (μM)
[Arsenite] (μM)
G24
H13
WT-HA
C13
E09
B24
M08
Het-HA
C15
F02
D18
J13
Hom-HA

Figure S6 – CFAP410 variants increase vulnerability to stress in ESCs and NPCs

Related to Figure 4. Kill curves of etoposide, valinomycin or sodium arsenite treated CFAP410 variants ESCs (a) or NPCs (b). Viability determined after 24h. Three experimental replicates performed with triplicate assay wells in each. Data presented as mean absorbance ±SEM. All X axes in log scale. Drug concentrations: Etoposide & Valinomycin 100.00, 33.33, 11.11, 3.70, 1.23, 0.41, 0.14 & 0.00µM, Sodium arsenite 30.00, 10.00, 3.33, 1.11, 0.37, 0.12, 0.04, 0.00µM.

Key to genotypes: WT-CFAP410$^{WT/WT}$: (R1); WT-HA: CFAP410$^{WT\text{-}HA/WT\text{-}HA}$ (G24 & H13); Het-HA: CFAP410$^{WT/V20M\text{-}HA}$ (C13 & E09) and CFAP410$^{WT/R73P\text{-}HA}$ (B24 & M08); Hom-HA: CFAP410$^{V20M\text{-}HA/V20M\text{-}HA}$ (C15 & F02) and CFAP410$^{R73P\text{-}HA/R73P\text{-}HA}$ (D18 & J13).

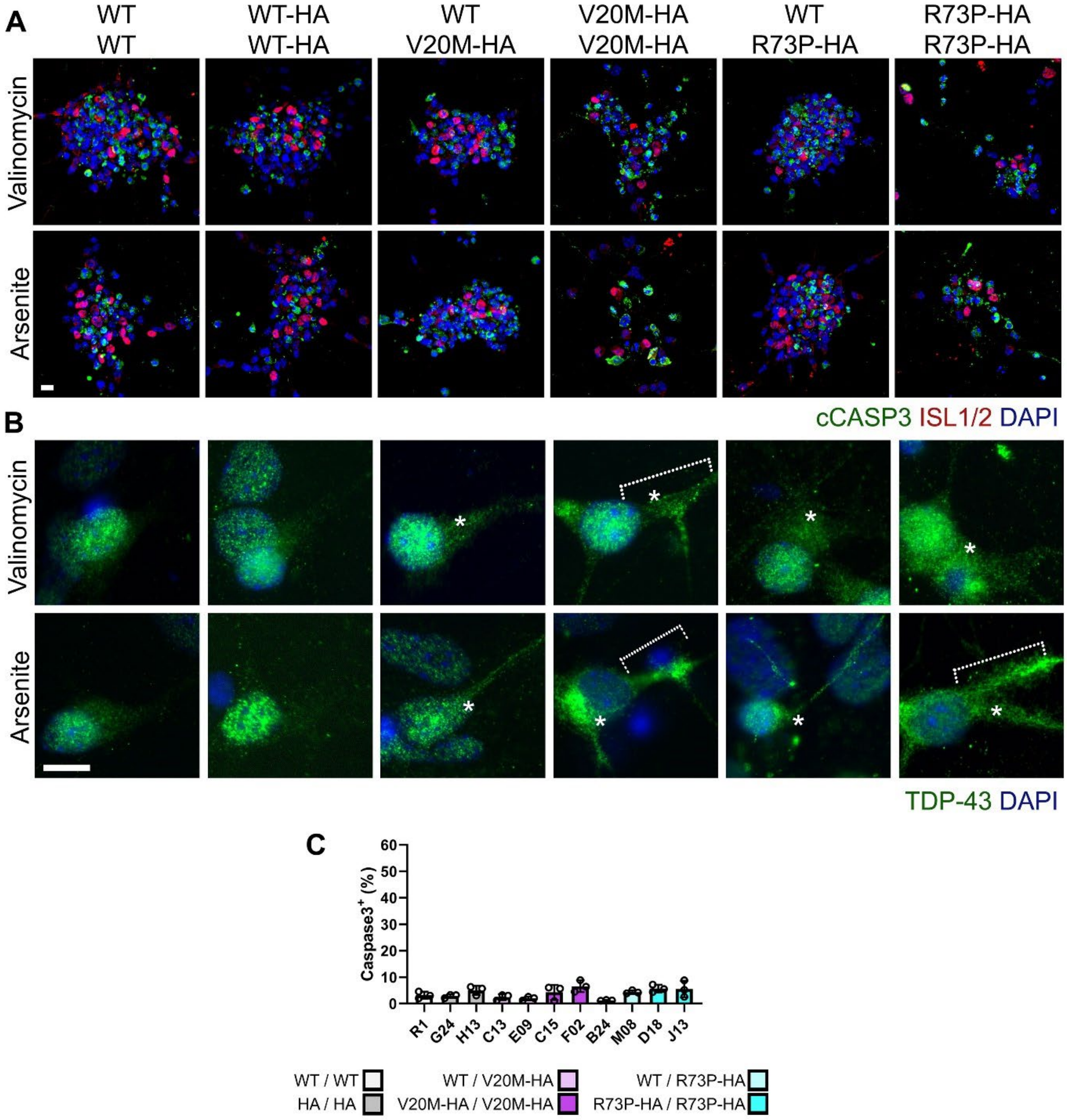
A
WT WT
WT-HA WT-HA
WT V20M-HA
V20M-HA V20M-HA
WT R73P-HA
R73P-HA R73P-HA
Valinomycin
Arsenite
cCASP3 ISL1/2 DAPI
B
Valinomycin
Arsenite
TDP-43 DAPI
C
Caspase3+ (%)
60
50
40
30
20
10
0
R1
G24
H13
C13
E09
C15
F02
B24
M08
D18
J13
WT / WT
HA / HA
WT / V20M-HA
V20M-HA / V20M-HA
WT / R73P-HA
R73P-HA / R73P-HA

Figure S7 – Cleaved caspase 3 and TDP-43 redistribution in CFAP410 variant neurons.

Related to Figure 5. Immunostaining for cleaved caspase 3 (cCASP2) and ISL1/2 (A), or TDP-43 (B) in neurons differentiated from CFAP410 variant ESCs and treated with valinomycin or sodium arsenite for 24h. Representative images from a single clone of each genotype shown. Scale bar 10μm. Representative immunostaining for graphs presented for arsenite and valinomycin in figure 6.

Average median cleaved caspase 3 positive nuclei frequency was quantified from four random fields each from three experiments (Bar represent mean ±SEM, data points represent experimental medians) without drug treatment (C).

Key to genotypes: WT/WT, $CFAP410^{WT/WT}$ (R1); HA/HA, $CFAP410^{WT\text{-}HA/WT\text{-}HA}$ (G24 & H13); WT/V20M-HA, $CFAP410^{WT/V20M\text{-}HA}$(C13 & E09); WT/R73P-HA, $CFAP410^{WT/R73P\text{-}HA}$ (B24 & M08); V20M-HA/V20M-HA, $CFAP410^{V20M\text{-}HA/V20M\text{-}HA}$ (C15 & F02); R73P-HA/R73P-HA, $CFAP410^{R73P\text{-}HA/R73P\text{-}HA}$ (D18 & J13).

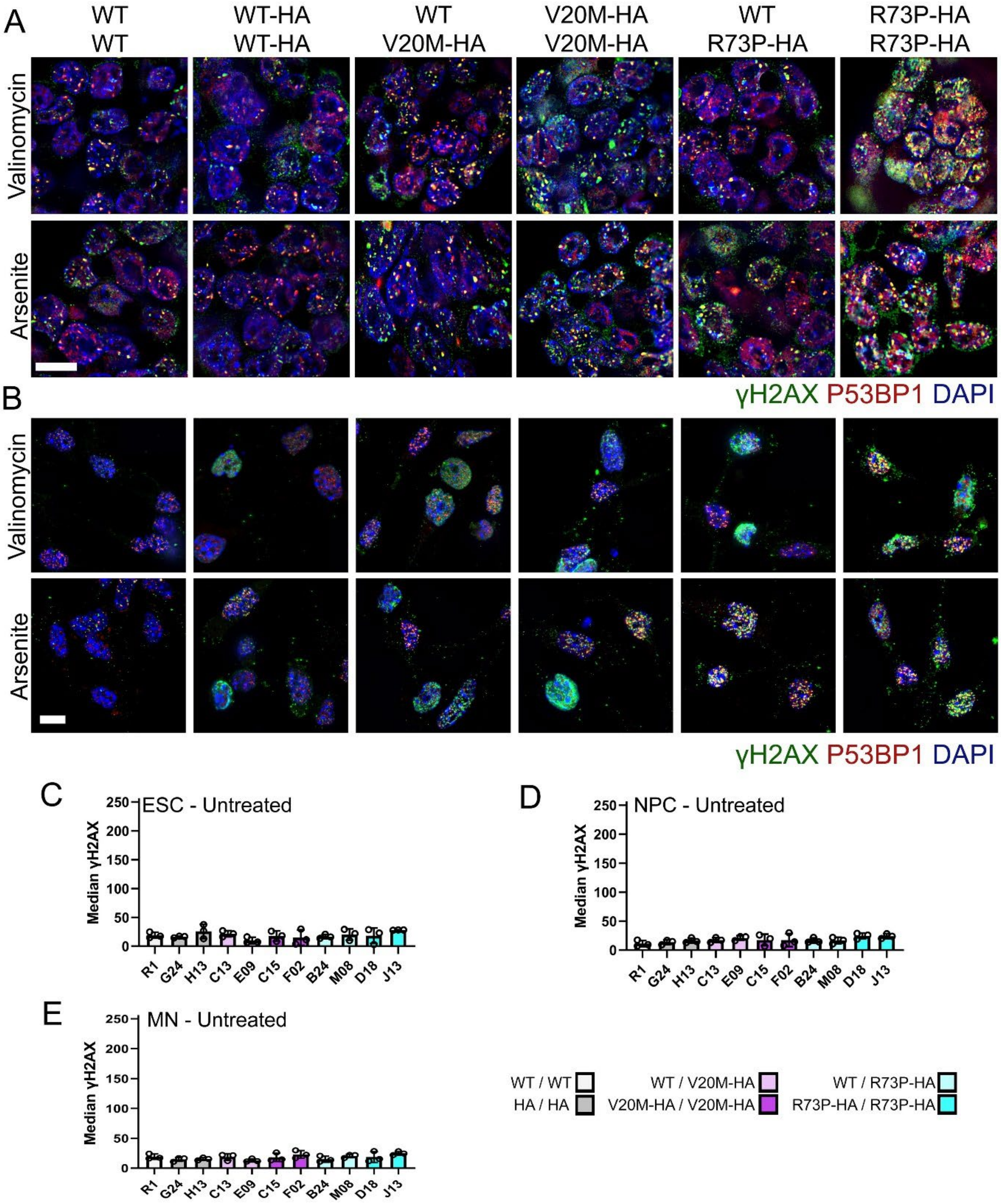

A
WT
WT
WT-HA
WT-HA
WT
V20M-HA
V20M-HA
V20M-HA
WT
R73P-HA
R73P-HA
R73P-HA
Valinomycin
Arsenite
γH2AX P53BP1 DAPI
B
Valinomycin
Arsenite
γH2AX P53BP1 DAPI
C
ESC - Untreated
Median γH2AX
250
200
150
100
50
0
R1 G24 H13 C13 E09 C15 F02 B24 M08 D18 J13
D
NPC - Untreated
Median γH2AX
250
200
150
100
50
0
R1 G24 H13 C13 E09 C15 F02 B24 M08 D18 J13
E
MN - Untreated
Median γH2AX
250
200
150
100
50
0
R1 G24 H13 C13 E09 C15 F02 B24 M08 D18 J13
WT / WT
HA / HA
WT / V20M-HA
V20M-HA / V20M-HA
WT / R73P-HA
R73P-HA / R73P-HA

Figure S8 – ESC and NPC lines carrying CFAP410 variants show increased DNA damage response.

Related to Figure 4. Immunostaining for γH2AX and P53BP1 in CFAP410 variant ESCs (A) and NPCs (B) treated with valinomycin or sodium arsenite for 24h. Representative images from a single clone of each genotype shown. Scale bar 10μm. Average median nuclear γH2AX intensity in ESC was quantified from four random fields each from three experiments (Bars represent mean ±SEM, data points represent experimental medians) without drug treatment in ESCs (C), NPCs (D) or MNs (E).

Key to genotypes: WT/WT, CFAP410$^{WT/WT}$ (R1); HA/HA, CFAP410$^{WT\text{-}HA/WT\text{-}HA}$ (G24 & H13); WT/V20M-HA, CFAP410$^{WT/V20M\text{-}HA}$(C13 & E09); WT/R73P-HA, CFAP410$^{WT/R73P\text{-}HA}$ (B24 & M08); V20M-HA/V20M-HA, CFAP410$^{V20M\text{-}HA/V20M\text{-}HA}$ (C15 & F02); R73P-HA/R73P-HA, CFAP410$^{R73P\text{-}HA/R73P\text{-}HA}$ (D18 & J13).

Table S1 - PCR Primers

Primers used for the T7E1 assay with Q5 HiFi Taq (NEB), High resolution melt curve analysis edited clones with GoTaq (Promega) and EvaGreen (Biotium), or in qPCR with iQ5 mix (BioRad).

Name	Sequence (5'-3')	Ta°C	Taq/mix	Product (bp)
HA tag R	AGCGTAATCTGGAACATCGTATGGGTA			
mC21 V20M T7E1 F1	CCTTCCCGACTCGACCTTTTCT	68	Q5	596
mC21 V20M T7E1 R1	AGTGAGCCTGCAACCTAGCTAG			
mC21 R73P T7E1 F1	TGCAGTGTCAACAGTGTCTCCA	68	Q5	308
mC21 R73P T7E1 R1	CCTCCTCAGTGGTTCCTTCAGG			
mC21 HA T7E1 F1	AGAGGTCAGGGGTCAGAAGCTA	68	Q5	507
mC21 HA T7E1 R1	CCTGCTTTGAAGGCTTTCCAGG			
mC21 V20M HRM F1	ACGAAAGATGGTCCTGTCCC	62	GoTaq +EvaGreen	148
mC21 V20M HRM R1	GAGTGAGCCTGCAACCTAGC			
mC21 R73P HRM F1	TCCCTGACAGTGTGCTGGTC	62	GoTaq +EvaGreen	213
mC21 R73P HRM R1	GTCATGCGGTAAAGGTGCG			
mC21_Rik20 1-F1	CCTACGCCCTCAACTCTGTC	62	iQ SYBRgreen	200
mC21_Rik20 1-R1	TGGCAGTCAGGATGTTCCTA			
mC21_Rik20 2-F1	AGAGAAGGTGCAGGCAATGG	62	iQ SYBRgreen	185
mC21_Rik20 2-R1	CAGGTGACATCCCTCTCCCTA			

mYWHAZ-F1	TTGTAGGAGCCCGTAGGTCA	62	iQ SYBRgreen	193
mYWHAZ-R1	TTGCTTTCTGGTTGCGAAGC			
Nestin-F1	CCCTGAAGTCGAGGAGCTG	62	iQ SYBRgreen	166
Nestin-R1	CTGCTGCACCTCTAAGCGA			
Pax6-F1	TAGCCCAGTATAAACGGGAGTG	62	iQ SYBRgreen	132
Pax6-R1	CCAGGTTGCGAAGAACTCTG			
Sox2-F1	GCGGAGTGGAAACTTTTGTCC	62	iQ SYBRgreen	157
Sox2-R1	CGGGAAGCGTGTACTTATCCTT			

Table S2 - Guide sequences

Forward and reverse sgRNA guide sequence oligos designed to anneal with overhangs for use with pX330 A1x2 and S2. Individual guides indicated by Guide ID number while pairs of guides for use with nickase Cas9 indicated by Pair ID. 'First cloned into' indicates first stage vector used prior to combination into a single vector carrying both guides. Bolded pairs selected for use in mESC editing.

Pair ID	Guide ID	Guide sequence (5'-3')	Cloning oligos (5'-3')	First cloned into
C21-HA_1a	10	GCCTGTGCAGCGCCTGTAGC	F: caccGCCTGTGCAGCGCCTGTAGC<br>R: aaacGCTACAGGCGCTGCACAGGC	pX330 dCas9 A1x2
C21-HA_1b	11	ATTTCAGACGCCCCAGTATC	F: caccgATTTCAGACGCCCCAGTATC<br>R: aaacGATACTGGGGCGTCTGAAATc	pX330 S2
C21-HA_2a	16*	GAAATCACTCCATGTCTTCC	F: caccgGAAATCACTCCATGTCTTCC<br>R: aaacGGAAGACATGGAGTGATTTCc	pX330 dCas9 A1x2
C21-HA_2b	17	CTGGACCCTTTTATATCCTG	F: caccgCTGGACCCTTTTATATCCTG<br>R: aaacCAGGATATAAAAGGGTCCAGc	pX330 S2
C21-HA_3a	18*	TCCATGTCTTCCTGGGGCTC	F: caccgTCCATGTCTTCCTGGGGCTC<br>R: aaacGAGCCCCAGGAAGACATGGAc	pX330 dCas9 A1x2
C21-HA_3b	11	ATTTCAGACGCCCCAGTATC	F: caccgATTTCAGACGCCCCAGTATC<br>R: aaacGATACTGGGGCGTCTGAAATc	pX330 S2
C21-HA_4a	19*	AAATCACTCCATGTCTTCCT	F: caccgAAATCACTCCATGTCTTCCT<br>R: aaacAGGAAGACATGGAGTGATTTc	pX330 dCas9 A1x2
C21-HA_4b	17	CTGGACCCTTTTATATCCTG	F: caccgCTGGACCCTTTTATATCCTG<br>R: aaacCAGGATATAAAAGGGTCCAGc	pX330 S2

C21-V20M_1a	1	CCGAAGCCTTGGCTCGGGAC	F: caccgCCGAAGCCTTGGCTCGGGAC<br>R: aaacGTCCCGAGCCAAGGCTTCGGc	pX330 dCas9 A1x2
C21-V20M_1b	2	AACTGCTGGTGAGGCCGGCC	F: caccgAACTGCTGGTGAGGCCGGCC<br>R: aaacGGCCGGCCTCACCAGCAGTTc	pX330 S2
C21-V20M_2a	1	CCGAAGCCTTGGCTCGGGAC	F: caccgCCGAAGCCTTGGCTCGGGAC<br>R: aaacGTCCCGAGCCAAGGCTTCGGc	pX330 dCas9 A1x2
C21-V20M_2b	3	ACGTGCGGAAGCTCAACTGC	F: caccgACGTGCGGAAGCTCAACTGC<br>R: aaacGCAGTTGAGCTTCCGCACGTc	pX330 S2
C21-V20M_3a	4	CAGCTCCGAAGCCTTGGCTC	F: caccgCAGCTCCGAAGCCTTGGCTC<br>R: aaacGAGCCAAGGCTTCGGAGCTGc	pX330 dCas9 A1x2
C21-V20M_3b	3	ACGTGCGGAAGCTCAACTGC	F: caccgACGTGCGGAAGCTCAACTGC<br>R: aaacGCAGTTGAGCTTCCGCACGTc	pX330 S2
C21-V20M_4a	5	GTTGTGCAGCTCCGAAGCCT	F: caccgGTTGTGCAGCTCCGAAGCCT<br>R: aaacAGGCTTCGGAGCTGCACAACc	pX330 dCas9 A1x2
C21-V20M_4b	2	AACTGCTGGTGAGGCCGGCC	F: caccgAACTGCTGGTGAGGCCGGCC<br>R: aaacGGCCGGCCTCACCAGCAGTTc	pX330 S2
C21-R73P_1a	6	TGGGGATGCGGTTCCTCCTC	F: caccgTGGGGATGCGGTTCCTCCTC<br>R: aaacGAGGAGGAACCGCATCCCCAc	pX330 dCas9 A1x2
C21-R73P_1b	7	TGAAAGACCTGCCACACCTC	F: caccgTGAAAGACCTGCCACACCTC<br>R: aaacGAGGTGTGGCAGGTCTTTCAc	pX330 S2
C21-R73P_2a	6	TGGGGATGCGGTTCCTCCTC	F: caccgTGGGGATGCGGTTCCTCCTC<br>R: aaacGAGGAGGAACCGCATCCCCAc	pX330 dCas9 A1x2

C21-R73P_2b	8	GAAAGACCTGCCACACCTCA	F: caccGAAAGACCTGCCACACCTCA<br>R: aaacTGAGGTGTGGCAGGTCTTTC	pX330 S2
C21-R73P_3a	9	GCTCGTTCAGACTGGGGATG	F: caccGCTCGTTCAGACTGGGGATG<br>R: aaacCATCCCCAGTCTGAACGAGC	pX330 dCas9 A1x2
C21-R73P_3b	8	GAAAGACCTGCCACACCTCA	F: caccGAAAGACCTGCCACACCTCA<br>R: aaacTGAGGTGTGGCAGGTCTTTC	pX330 S2
C21-R73P_4a	9	GCTCGTTCAGACTGGGGATG	F: caccGCTCGTTCAGACTGGGGATG<br>R: aaacCATCCCCAGTCTGAACGAGC	pX330 dCas9 A1x2
C21-R73P_4b	7	TGAAAGACCTGCCACACCTC	F: caccgTGAAAGACCTGCCACACCTC<br>R: aaacGAGGTGTGGCAGGTCTTTCAc	pX330 S2

* highlighted cloning oligo pairs contain a BpiI site so were cloned into pX330 conventionally, rather than by GoldenGate

Table S3 – Gibson Assembly cloning primers

Primers with overlapping homology designed to amplify genomic sequences for assembly cloning into pBS puroDtk.

5’ R and 3’ F contain overlapping homology with desired point mutations. 5’F and 3’R contain pBS puroDtk homology

Name	Sequence (5'-3')	Ta ('C)
C21 HA 5' hom arm F	CACTATAGGGCGAATTGGAGCTCCACCGCGGGGCAGCAAGGAGAAAGGGTTG	72
C21 HA 5' hom arm R	GGTAGAACAGCTCGTTAAGACTGGGGATGGGATTCCTCCTCAGATACAGCTCGC	
C21 HA 3' hom arm F	GCGAGCTGTATCTGAGGAGGAATCCCATCCCCAGTCTTAACGAGCTGTTCTACC	72
C21 HA 3' hom arm R	TCCACTAGTTCTAGAGCGGCCGCCACCGCGCCCAGGGCCTTGCAAATGCTAG	
C21 V20M 5' hom arm F	CACTATAGGGCGAATTGGAGCTCCACCGCGGCCAGTCTTGCCAGAAACTGGCTCAGGCAATCC	72
C21 V20M 5' hom arm R	CCGGCCTCATCAGCAGTTAAGCTTCCGCATGTTGTGCAGCTCCGAAGCC	
C21 V20M 3' hom arm F	CACTATAGGGCGAATTGGAGCTCCACCGCGGCCAGTCTTGCCAGAAACTGGCTCAGGCAATCC	72
C21 V20M 3' hom arm R	GGCTTCGGAGCTGCACAACATGCGGAAGCTTAACTGCTGATGAGGCCGG	
C21 R73P 5' hom arm F	CACTATAGGGCGAATTGGAGCTCCACCGCGGGGCAGCAAGGAGAAAGGGTTG	72
C21 R73P 5' hom arm R	GGTAGAACAGCTCGTTAAGACTGGGGATGGGATTCCTCCTCAGATACAGCTCGC	
C21 R73P 3' hom arm F	TCCACTAGTTCTAGAGCGGCCGCCACCGCGCCCAGGGCCTTGCAAATGCTAG	72
C21 R73P 3' hom arm R	GCGAGCTGTATCTGAGGAGGAATCCCATCCCCAGTCTTAACGAGCTGTTCTACC	

Table S4 – Antibodies

Antibodies used in this study for immunostaining or Western blot (WB as indicated)

Name	Supplier	Cat#	Dilution
Islet1/2	DSHB	40.2D6	1:5
Pericentrin	Abcam	ab4448	1:500
Peripherin	Millipore	AB1530	1:1000
TDP43	ProteinTech	10782-2-AP	1:1000
Nestin	DSHB	Rat401	1:50
YH2AX	Biolegend	613401	1:1000 (1:5000 WB)
P53BP1	Novus	NB100-304	1:1000 (1:5000 WB)
HA	Covance	MMS-101P	1:1000
Cleaved caspase3	Abcam	AB13847	1:500
MitoTracker Red CMXRos	Invitrogen	M7512	50nM
Arl13B	Proteintech	17711-1-AP	1:500
NEK1	Santa cruz	sc398813	1:5000 (WB)
SPATA7	Proteintech	12020-1-ap	1:5000 (WB)
FBXO3	Santa cruz	514625	1:5000 (WB)
pCHK1 (Ser317)	Cell Signalling	54762S	1:1000 (WB)
CHK1	Santa Cruz	sc8408	1:1000 (WB)
HRP α GAPDH	Abcam	Ab9482	1:10,000 (WB)
AlexaFluor 488 Gt α Ms	Invitrogen	A-11001	1:2000
AlexaFluor 594 Gt α Rbt	Invitrogen	A-11008	1:2000
AlexaFluor 488 Gt α Rbt	Invitrogen	A-11012	1:2000
AlexaFluor 568 Gt α Rbt	Invitrogen	A-11011	1:2000
AlexaFluor 488 Gt α Rt	Invitrogen	A-11006	1:2000

Supplementary Data - Full gel images

Data S1 - Uncropped gel from Figure 1D

HA tag zygosity in HA edited clones was determined by PCR across the locus followed by EcoRI digest. Donor plasmid and R1 ESC parent DNA was used as mutant (Mt) and wildtype (WT) controls.

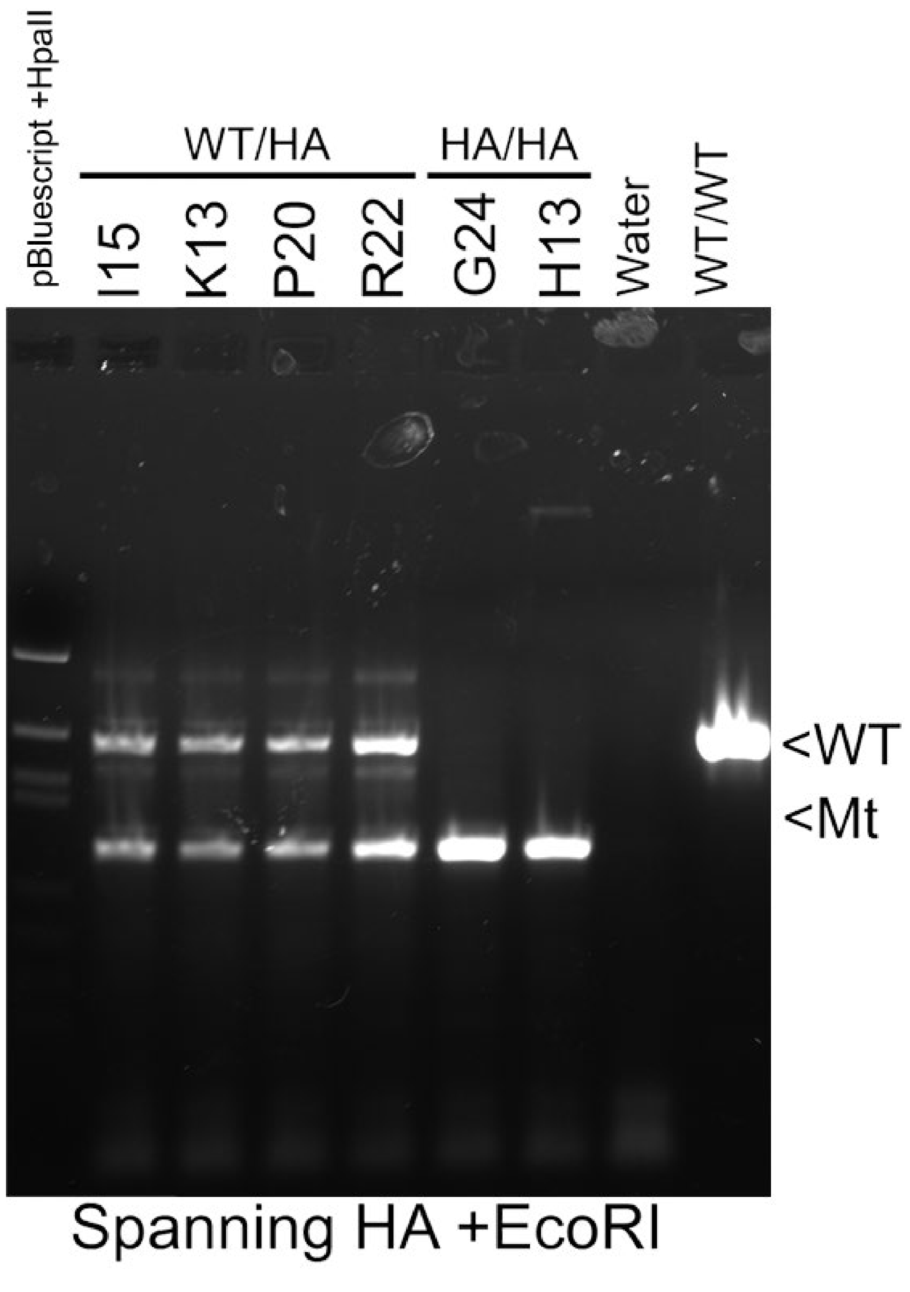

Data S2 - Uncropped gel from Figure 1E

V20M zygosity in HA het and hom edited clones was determined by PCR across the locus followed by MseI digest. Donor plasmid and R1 ESC parent DNA was used as mutant (Mt) and wildtype (WT) controls.

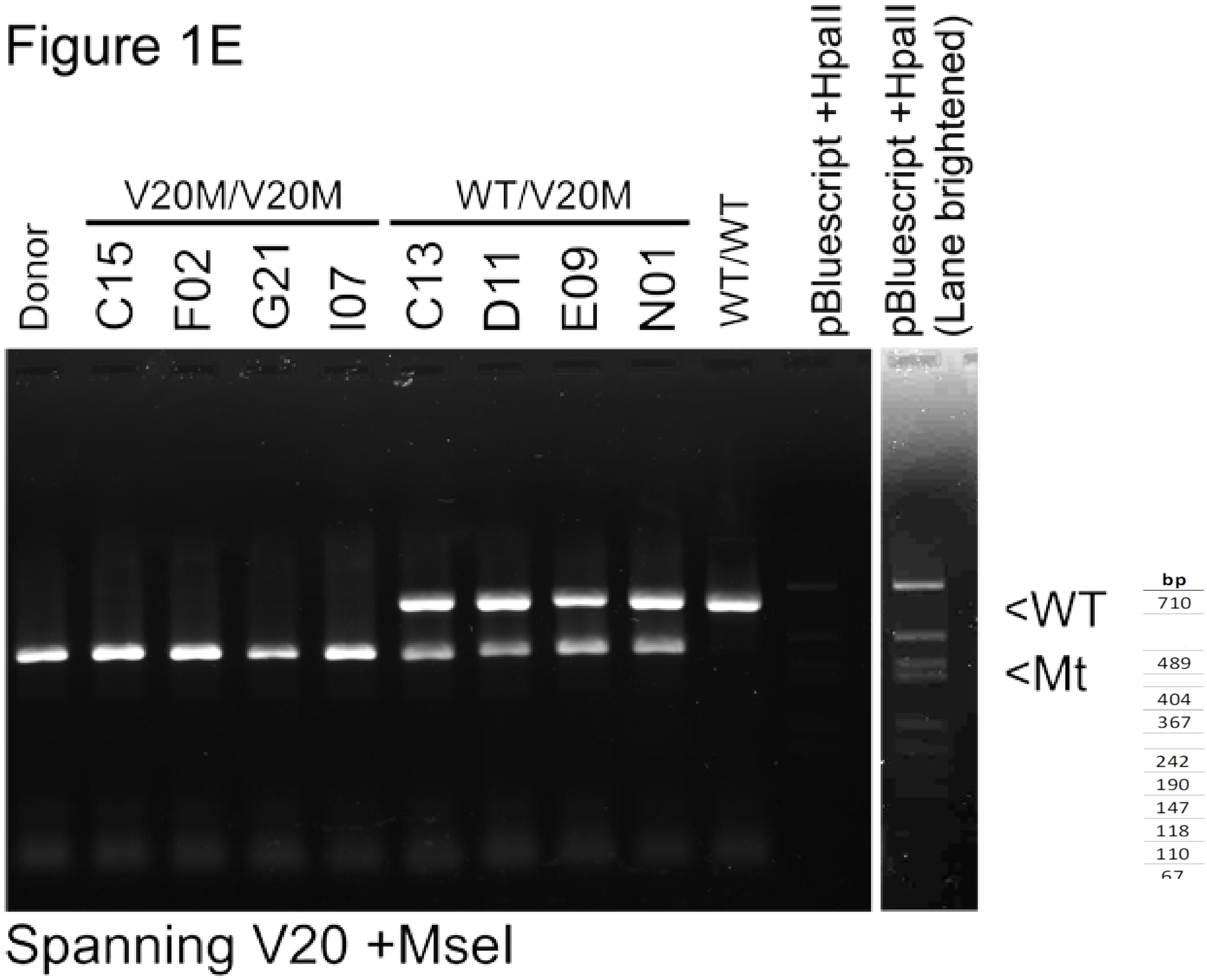

Data S3 - Uncropped gel from Figure 1G

R73P zygosity in HA het and hom edited clones was determined by PCR across the locus followed by MseI digest. Donor plasmid and R1 ESC parent DNA was used as mutant (Mt) and wildtype (WT) controls.

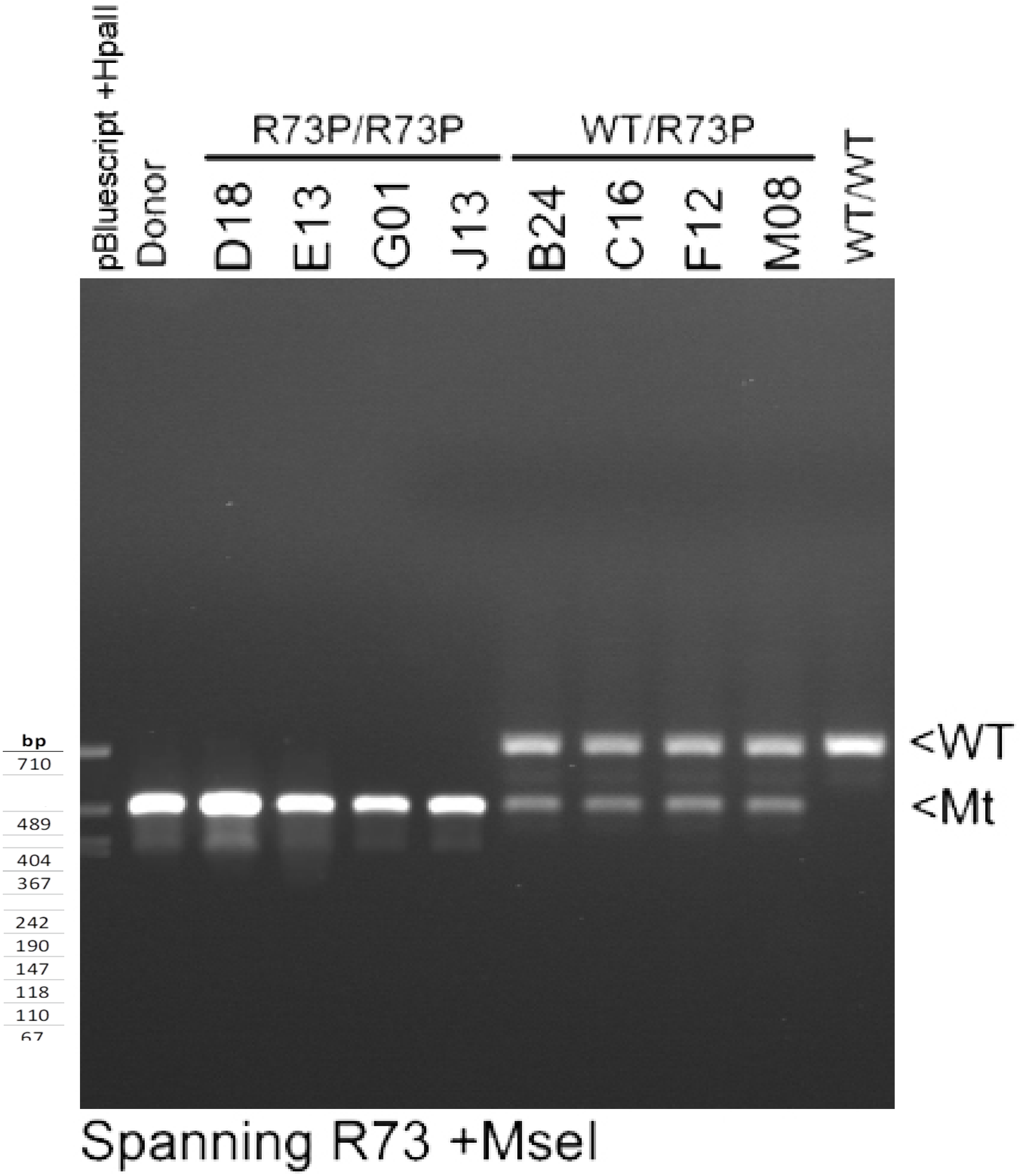

Data S4 - Uncropped gel from Figure 1H

To determine whether heterozygous V20M or R73P mutations were present in the HA tagged allele, RT-PCR was performed on HA-primed cDNA followed by MseI digest (h). Successful digestion indicated the point mutations were present on HA tagged allele.

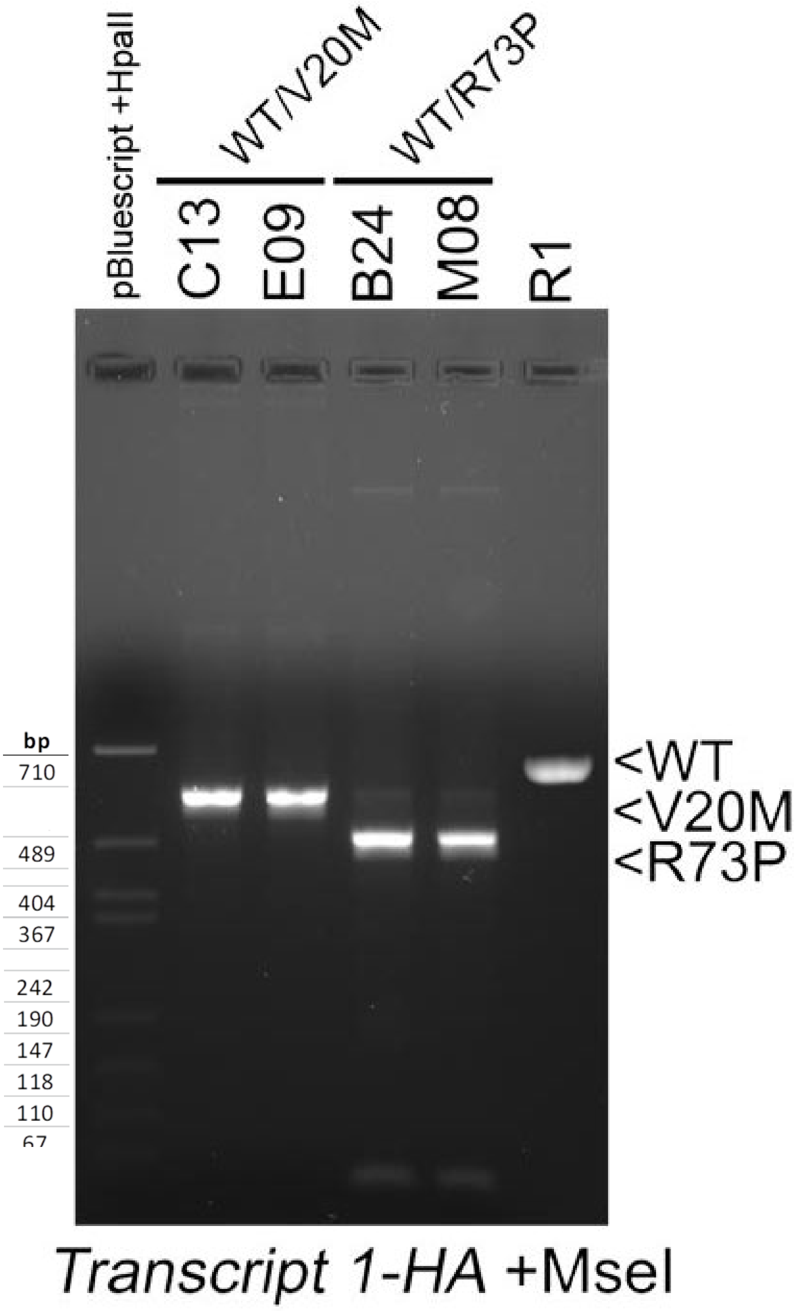

Data S5 - Uncropped gel from Figure 1E

Western blot to show CFAP410-HA expression immunoblotted using anti-HA antibody (e). GAPDH as loading control. Transgenic R1 overexpressing HA tagged human CFAP410 used as a positive control.

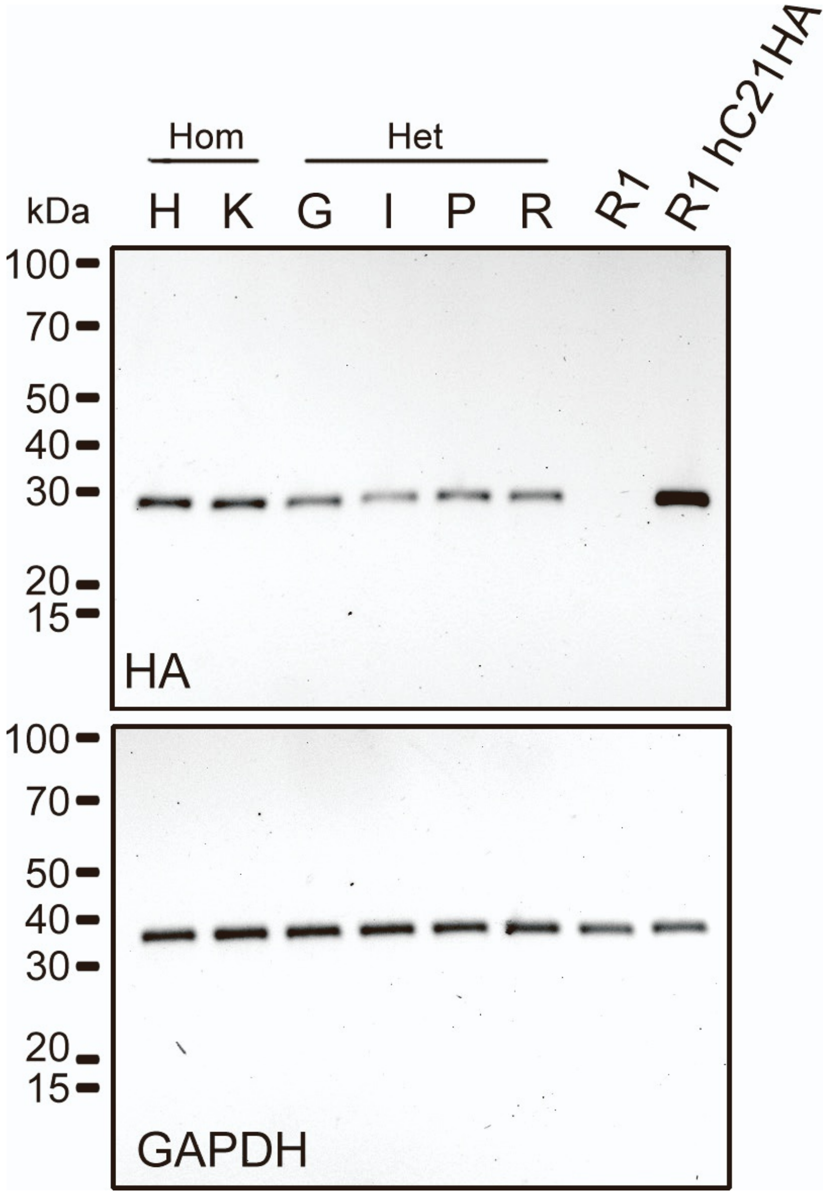

Data S6 - Uncropped gel from Figure 2 A & B

Western blot of two representative clones from each genotype immunoblotted using anti-HA antibody (a). GAPDH as loading control.

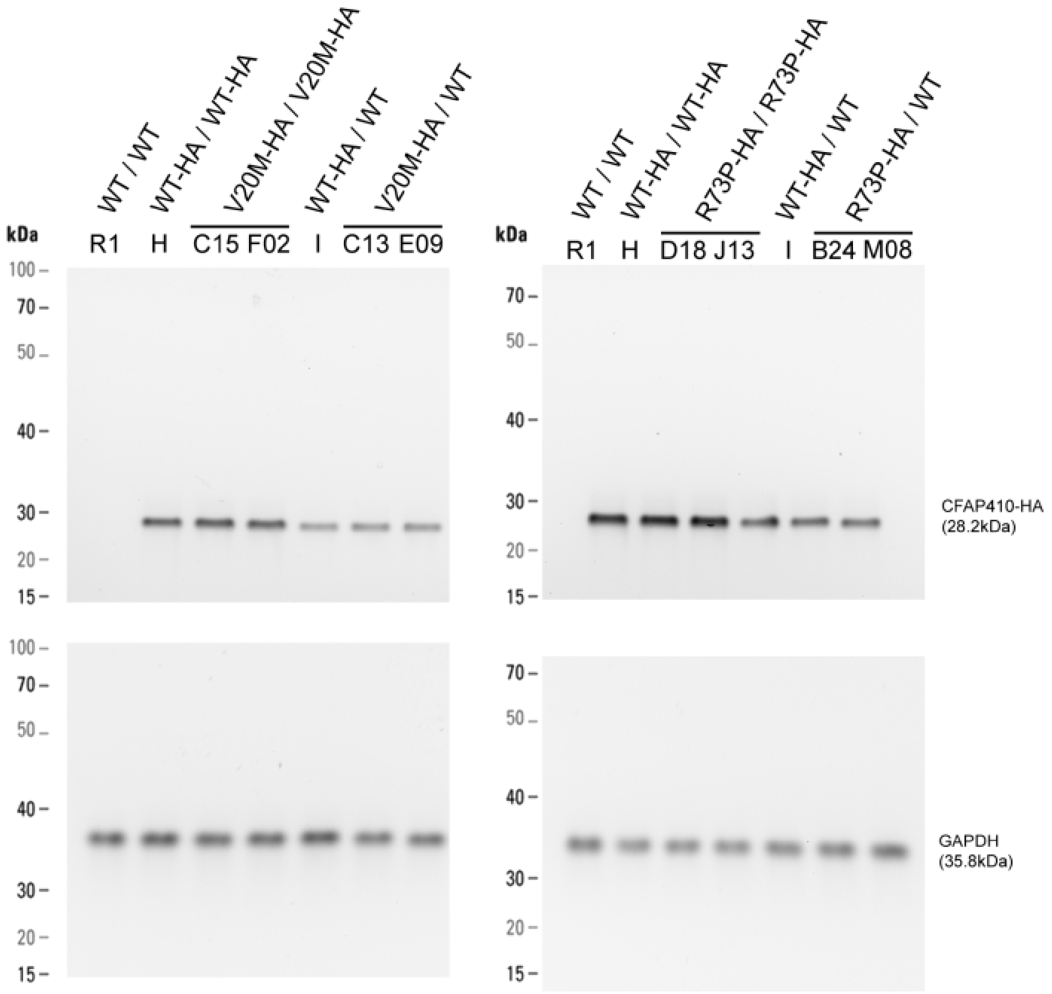

Data S7 - Uncropped gel from Figure 2D

Co-immunoprecipitation using antibody against the HA tag and immunoblotting for NEK1, SPATA7 and FBXO3. Performed on neuronal culture lysates of R1 (CFAP410$^{WT/WT}$), G24 & H13 (CFAP410$^{WT\text{-}HA/WT\text{-}HA}$), C15 & F02 (CFAP410$^{V20M\text{-}HA/V20M\text{-}HA}$), D18 & J13 (CFAP410$^{R73P\text{-}HA/R73P\text{-}HA}$).

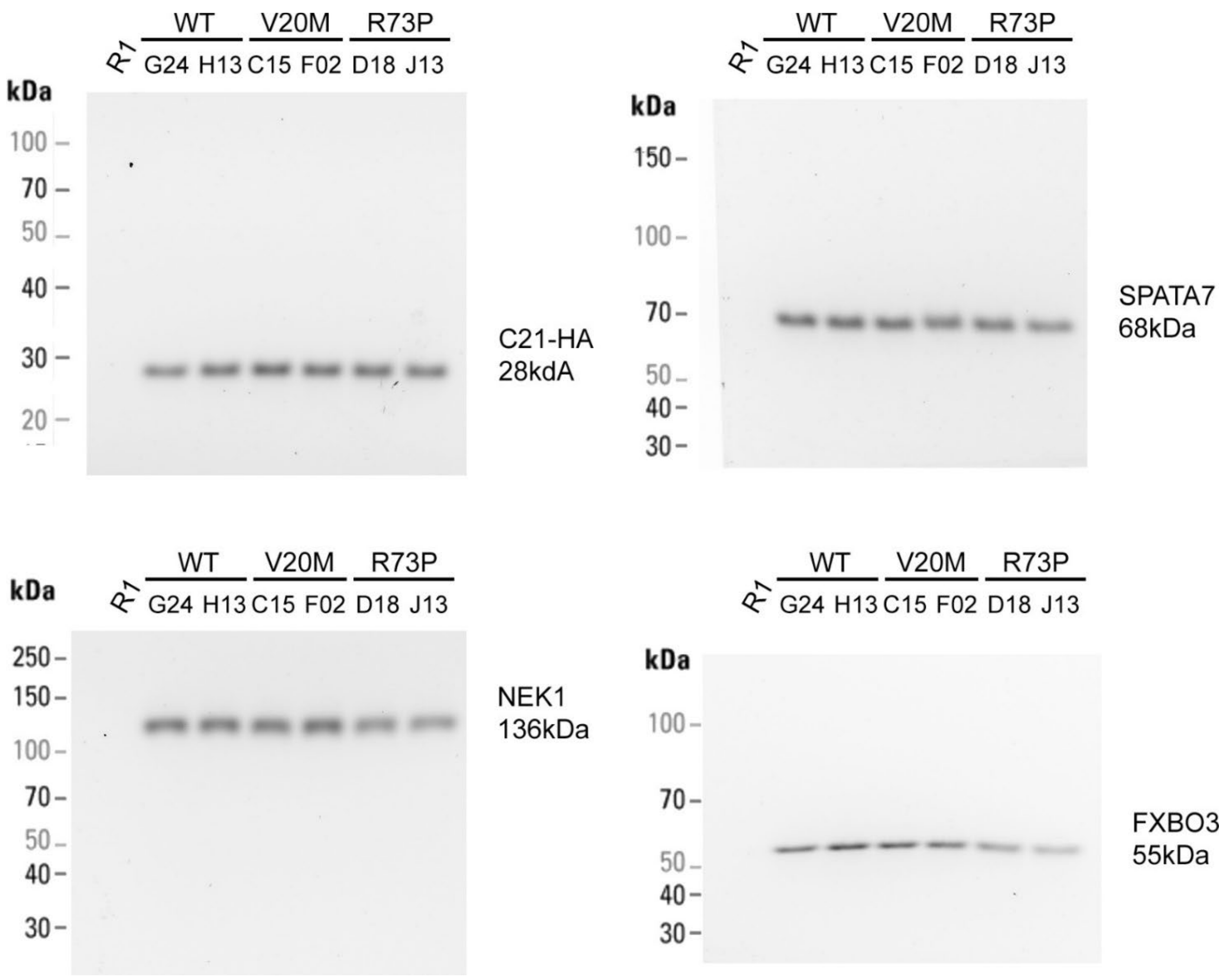

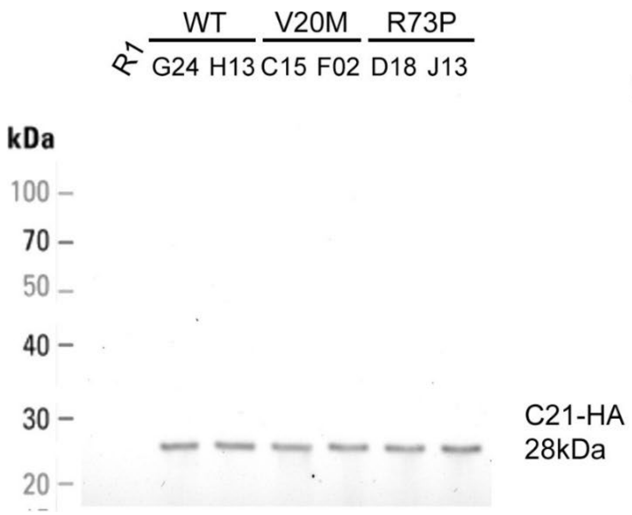
WT
V20M
R73P
R1
G24
H13
C15
F02
D18
J13
kDa
100
70
50
40
30
20
C21-HA
28kDa

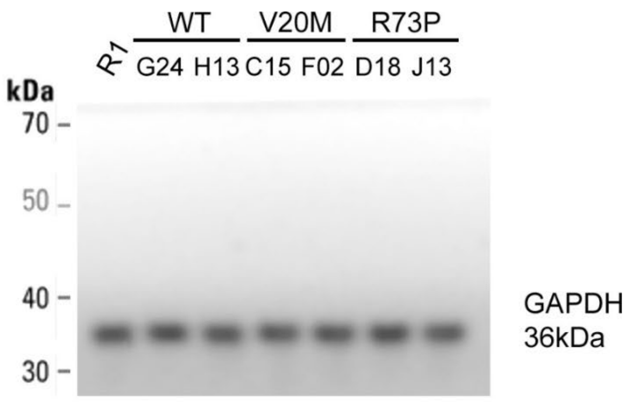
WT
V20M
R73P
R1
G24
H13
C15
F02
D18
J13
kDa
70
50
40
30
GAPDH
36kDa

Data S8 - Uncropped gel from Figure 8E

Neurons differentiated from CFAP410 variant ESCs and treated with etoposide, valinomycin or sodium arsenite for 24h. Representative western blot of untreated and etoposide treated cells for γH2AX, P53BP1, phospho & total CHK1 (e). GAPDH loading control.

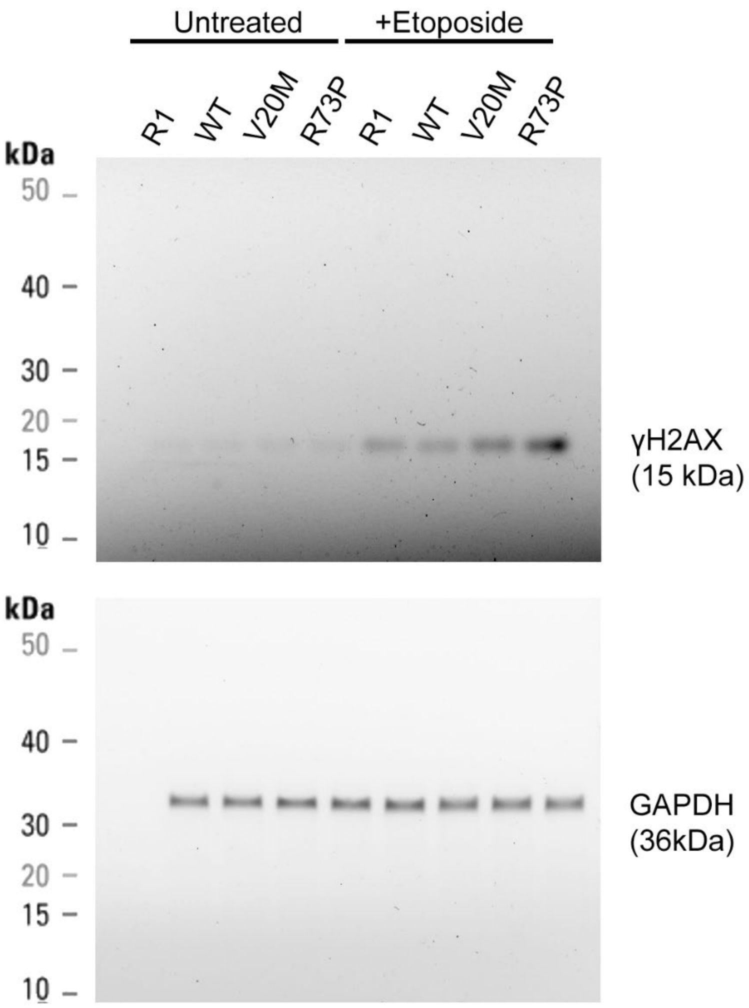

Supplement: Document S1. Figures S1–S8, Tables S1–S4, and Data S1–S8 [file mmc1.pdf]
